# Analysis of the *Rana catesbeiana *tadpole tail fin proteome and phosphoproteome during T_3_-induced apoptosis: identification of a novel type I keratin

**DOI:** 10.1186/1471-213X-7-94

**Published:** 2007-08-06

**Authors:** Dominik Domanski, Caren C Helbing

**Affiliations:** 1 Department of Biochemistry & Microbiology, University of Victoria, PO Box 3055, Victoria, BC V8W 3P6, Canada

## Abstract

**Background:**

Thyroid hormones (THs) are vital in the maintenance of homeostasis and in the control of development. One postembryonic developmental process that is principally regulated by THs is amphibian metamorphosis. This process has been intensively studied at the genomic level yet very little information at the proteomic level exists. In addition, there is increasing evidence that changes in the phosphoproteome influence TH action.

**Results:**

Here we identify components of the proteome and phosphoproteome in the tail fin that changed within 48 h of exposure of premetamorphic *Rana catesbeiana *tadpoles to 10 nM 3,5,3'-triiodothyronine (T_3_). To this end, we developed a cell and protein fractionation method combined with two-dimensional gel electrophoresis and phosphoprotein-specific staining. Altered proteins were identified using mass spectrometry (MS). We identified and cloned a novel Rana larval type I keratin, RLK I, which  may be a target for caspase-mediated proteolysis upon exposure to T_3_. In addition, the RLK I transcript is reduced during T_3_-induced and natural metamorphosis which is consistent with a larval keratin. Furthermore, GILT, a protein involved in the immune system, is changed in phosphorylation state which is linked to its activation. Using a complementary MS technique for the analysis of differentially-expressed proteins, isobaric tags for relative and absolute quantitation (iTRAQ) revealed 15 additional proteins whose levels were altered upon T_3 _treatment. The success of identifying proteins whose levels changed upon T_3 _treatment with iTRAQ was enhanced through *de novo *sequencing of MS data and homology database searching. These proteins are involved in apoptosis, extracellular matrix structure, immune system, metabolism, mechanical function, and oxygen transport.

**Conclusion:**

We have demonstrated the ability to derive proteomics-based information from a model species for postembryonic development for which no genome information is currently available. The present study identifies proteins whose levels and/or phosphorylation states are altered within 48 h of the induction of tadpole tail regression prior to overt remodeling of the tail. In particular, we have identified a novel keratin that is a target for T_3_-mediated changes in the tail that can serve as an indicator of early response to this hormone.

## Background

Thyroid hormones (TH) are important signaling molecules in vertebrates that regulate homeostasis, growth, and development. One developmental process that is dependent upon the presence of TH is amphibian metamorphosis. During metamorphosis the larval, aquatic, herbivorous tadpole transforms into a terrestrial, carnivorous juvenile frog. This event requires drastic changes in essentially every organ and tissue of the tadpole and includes: the resorption of larval organs and tissues, remodeling of larval organs into juvenile form, and *de novo *development of organs and tissues [1]. Metamorphosis is completely controlled through the control of serum TH levels. The thyroid gland mainly produces the thyroid hormone, 3,5,3',5'-tetraiodothyronine (T_4 _or thyroxine), which is converted into the biologically more active form, 3,5,3'-triiodothyronine (T_3_), in the peripheral tissues [1,2]. Progression through natural metamorphosis is dependent upon a tightly-controlled rise and fall in TH levels. Premetamorphic tadpoles are functionally athyroid with no measurable levels of THs [3]. TH levels gradually increase during prometamorphosis and reach maximal levels at metamorphic climax. At this stage, overt remodelling of the tadpole rapidly ensues. Premetamorphic tadpoles can be induced to undergo precocious metamorphosis by exposure to TH [1].

The best understood mechanism of TH action involves TH binding to nuclear thyroid hormone receptors (TRs) which regulate gene expression [2,4]. TH binding to TRs can either activate or repress transcription of responsive genes through recruitment of coactivators and corepressors, respectively [2,4]. Differential nuclear TR levels and intracellular T_3 _levels controlled by deiodinase activity and TH-binding proteins, contribute to tissue-specific responses [1,2,4]. However, the response to TH also depends on the existing complement of other proteins that can influence cell fate (e.g. regression of the tail *versus *growth and differentiation of the hindlimb). However, the molecular mechanisms are poorly understood.

Most TH-responsive genes are up-regulated and these have been most commonly studied particularly in the tail [5-10]. The genetic program required for tail regression is established between 24 and 48 h of TH exposure at a "commitment point" after which removal of TH or exposure to transcription or protein synthesis inhibitors cannot prevent regression [5,11]. Studies based on PCR subtractive hybridization methods and cDNA gene arrays have identified a number of possible genes involved in this process [5-9]. However, the relationship of most of these genomic findings to changes in the proteome has yet to be identified and there is growing evidence for non-classical TH action through phosphorylation signaling pathways [12-16].

Proteomic scale studies on TH-dependent tail regression have been scarce. Ray *et al. *identified several ^35^S-methionine labelled proteins from *Rana catesbeiana *tail fin that change during natural and precocious metamorphosis using two-dimensional (2D) gel analysis [17]. Kobayashi *et al. *used 2D gel electrophoresis to analyze changes in protein expression in the back and tail skin of *Xenopus laevis *during metamorphosis [18]. From the 2D protein spot patterns they could classify the back skin into larval or adult type and observe the transition. Attempts were made in these studies to identify the altered protein spots. This, however, involved identifying the spots based on position, comigration or immunological detection methods. Using 2D gel separation and mass spectrometry (MS) for protein identification, we were able to identify 9 proteins whose expression was altered in the *X. laevis *tadpole tail during TH-induced metamorphosis [6]. Regardless of the approach, the lack of any sample fractionation led to the identification of only abundant proteins.

In this work we identified novel changes in the proteome and phosphoproteome associated with the induction of T_3_-dependent tail regression of *Rana catesbeiana *tadpoles. Proteins that changed in abundance were detected using two-dimensional (2D) gel electrophoresis and isobaric tags for relative and absolute quantitation (iTRAQ) methods. Alterations in phosphorylation were revealed using a phosphoprotein-specific stain. Mass spectrometry (MS) analyses were then used for protein identification. Proteome coverage was enhanced through the use of cell and protein fractionation to reveal several proteins that are altered within 48 h of T_3 _exposure.

## Results and discussion

### Fractionation of the tail fin proteome and 2D gel analyses

The subcellular fractionation method was optimized to provide highly enriched fractions of cytosolic, mitochondrial, nuclear and microsomal samples from the *R. catesbeiana *tail fin. The goals of the method were to limit sample complexity with effective cell fractionation with minimal cross-contamination and reasonable yields. The fractions also had to be salt and buffer-compatible with subsequent proteomic analysis.

**Figure 1:**
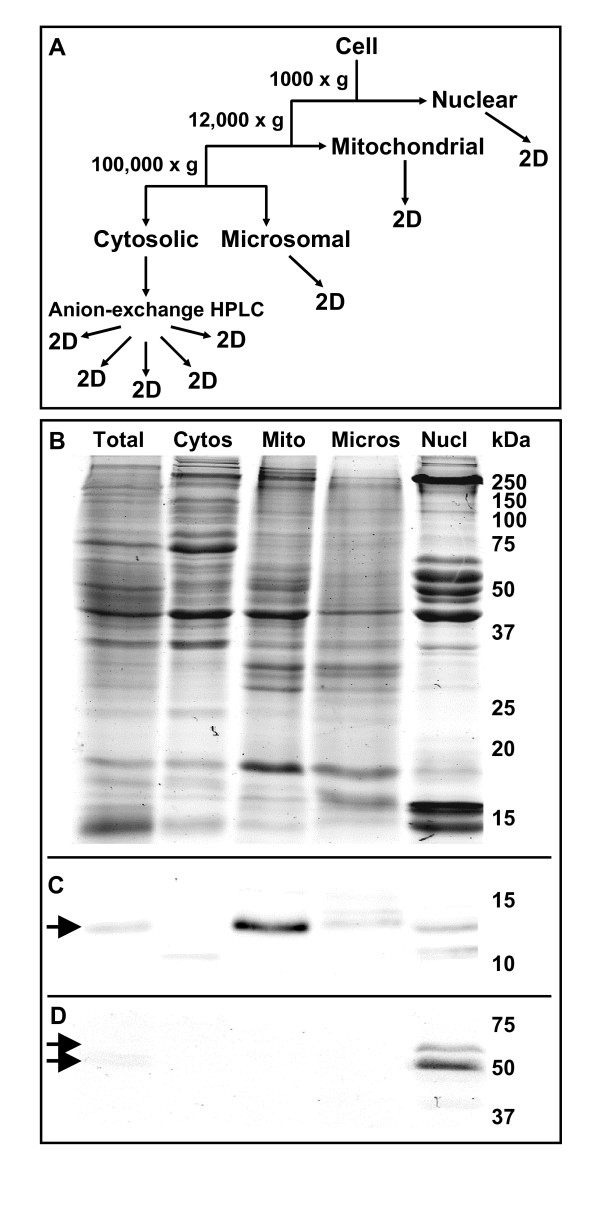
**Subcellular fractionation of the tail fin proteome**. (A) Fractionation of tail fin cells into subcellular compartments and subsequent treatments of those fractions. Two different extraction procedures, based on differential centrifugation, were developed to generate the nuclear and the cytoplasmic/mitochondrial/microsomal fractions. (B) SDS-PAGE shows the successful fractionation of the total tail fin proteome into the cytosolic (Cytos), mitochondrial (Mito), microsomal (Micros), and nuclear (Nucl) fractions. Relative molecular weights of protein standards are indicated in kDa. (C) Immunoblot of the gel in (B) for the mitochondrial marker, cytochrome c (arrow) showing the enrichment of mitochondria in the expected fraction. (D) Immunoblot of the gel in (B) for the nuclear markers, lamin B1 and B2 (double arrow) showing the enrichment of nuclei in the expected fraction.

We developed two separate procedures based on differential centrifugation to generate nuclear and cytosolic/mitochondrial/microsomal fractions (Fig. 1A). The nuclear extraction procedure was optimized to minimize nuclear clumping, increase nuclei stability, and minimize cytoskeletal, cytoplasmic/organelle and DNA contamination. The cytosolic/mitochondrial/microsomal extraction procedure was developed to increase the disruption of tail fin cell membrane and increase mitochondrial stability and purity. The mitochondria-enriched fraction was obtained with a 12,000 × g centrifugation and it likely also contained lysosomes, peroxisomes, Golgi and endoplasmic reticulum (ER). Centrifugation at 100,000 × g removed the vesicles of the plasma membrane, endosomes, Golgi and ER into the microsomal pellet leaving the cytosolic supernatant with its soluble molecules (cytosolic fraction; Fig. 1B). Nuclear integrity was monitored by microscopy (data not shown) and fractionation efficiency was determined using immunoblot analysis for subcellular markers. An immunoblot for cytochrome c (a mitochondrial marker) shows substantial enrichment for that organelle in the mitochondrial fraction (Fig. 1C) while an immunoblot for the nuclear markers, lamins B1 and B2, shows enrichment of nuclei in the nuclear fraction (Fig. 1D).

**Figure 2:**
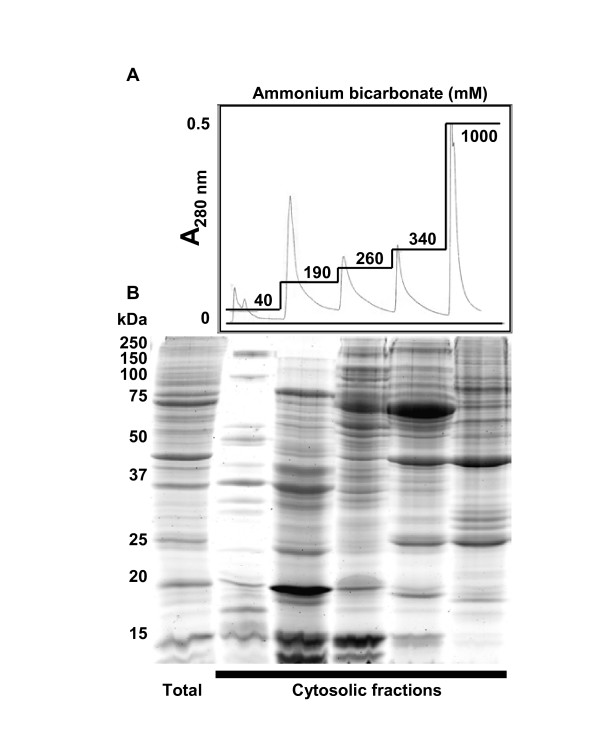
**Anion-exchange HPLC fractionation of the cytosolic fraction**. (A) The cytosolic fraction was further fractionated using an anion-exchange column (Accell QMA) with a step-gradient of increasing concentrations of ammonium bicarbonate (straight lines). The concentrations are indicated on each step while absorbance was measured at 280 nm indicating the protein yield of each fraction. (B) The Coomassie blue-stained SDS-PAGE gel shows the fractionation of the cytosolic sample (total) with the lanes corresponding to the cytosolic fractions below the peaks of the HPLC chromatogram. Note the resulting enrichment of certain protein bands. Relative molecular weights of protein standards are indicated in kDa.

**Figure 3:**
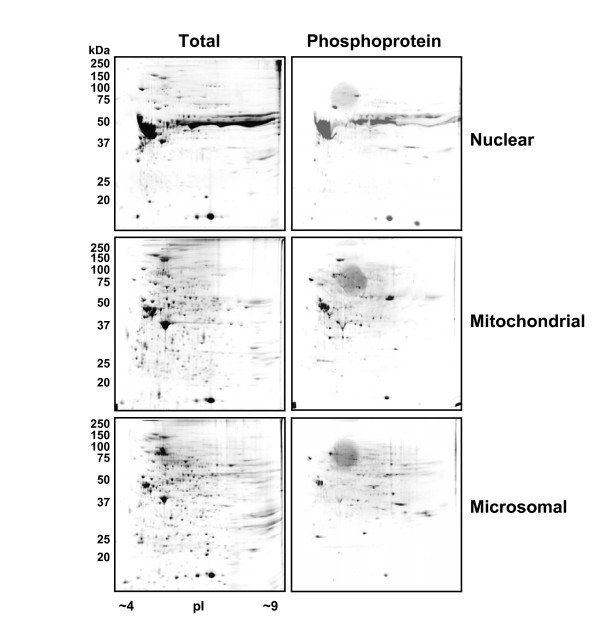
**2D gel analyses of the nuclear, mitochondrial and microsomal fractions**. Proteins from the nuclear, mitochondrial and microsomal fractions were separated by 2D-PAGE according to molecular weight and pI point. Total proteins were detected by colloidal Coomassie stain while phosphoproteins were detected in the same gel using the ProQ Diamond phosphoprotein-specific stain. Relative molecular weights of protein standards are indicated in kDa.

**Figure 4:**
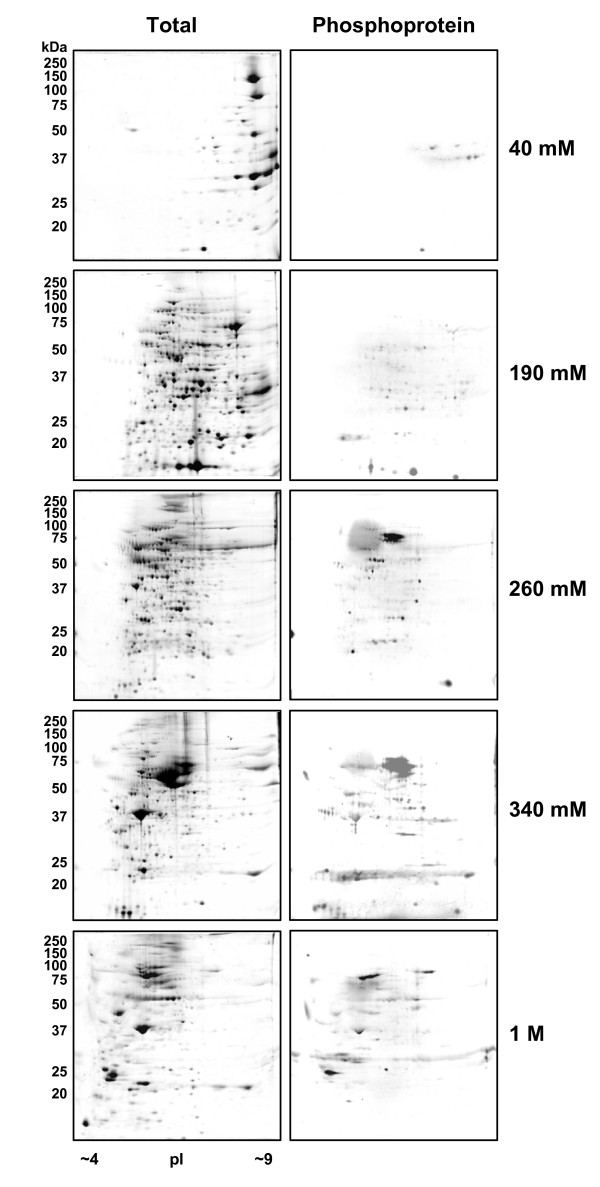
**2D gel analysis of the anion-exchange HPLC cytosolic fractions**. Proteins from each of the fractions resulting from the anion-exchange HPLC of the cytosolic sample were separated by 2D-PAGE according to molecular weight and pI point. The 40 mM fraction is the unbound protein fraction, while the subsequent fractions are proteins eluted by the increasing ammonium bicarbonate concentration step-gradients. Total proteins were detected by colloidal Coomassie stain while phosphoproteins were detected in the same gel using the ProQ Diamond phosphoprotein-specific stain. Relative molecular weights of protein standards are indicated in kDa.

The cytosolic fraction is a complex mixture of many proteins and was therefore further fractionated using anion-exchange high performance liquid chromatography (HPLC) (Fig. 2A). A step-gradient anion-exchange HPLC procedure was developed that used ammonium bicarbonate as a volatile buffer in place of commonly used salt and non-volatile buffer to provide salt-free fractions after lyophilization to render the samples compatible with the subsequent 2D gel analysis. The cytosolic fraction was thus further fractionated into five fractions: 40 mM (unbound proteins), 190 mM, 260 mM, 340 mM and 1 M ammonium bicarbonate with each fraction (except 40 mM) yielding roughly equal amounts of protein as shown by SDS-PAGE (Fig. 2B). Proteins within each of the resulting fractions were then separated by 2D polyacrylamide gel electrophoresis which separates proteins based on their molecular weight and isoelectric point (pI). Therefore, the entire fractionation protocol divided the tail fin proteome over eight 2D gels: nuclear, mitochondrial, microsomal (Fig. 3) and five cytosolic fractions (Fig. 4). This fractionation method increases the ability to observe expression changes in low abundance proteins and provides information on subcellular localization of proteins which cannot be achieved by examining a whole cell homogenate on a single 2D gel. From our results, it is evident that each of the fractions shows a distinctive pattern of spots with many unique spots per fraction analyzed (Figs. 3 and 4). There is some overlap with the more abundant protein spots between the neighboring fractions of the HPLC separation and between the microsomal and mitochondrial fractions, which probably share many cellular membrane compartments (e.g. Golgi, ER, and lysosomes). Phosphoproteins were detected on the 2D gels with a phosphoprotein-specific fluorescent stain (Pro-Q Diamond) which detects phosphorylation on Ser, Thr and Tyr residues [19] (Figs. 3 and 4). Total proteins and phosphoproteins were detected in the same 2D gel allowing for easy identification of phosphoprotein location and subsequent isolation for MS analysis.

The above methods were used to analyze the proteome and phosphoproteome of the premetamorphic *R. catesbeiana *tail fin undergoing precocious metamorphosis at 24 and 48 h induced with 10 nM T_3_. A minimum of three independent replicates allowed for the verification of changes in protein and phosphoprotein expression and MS analysis was used for protein identification.

### Identification of a unique *R. catesbeiana *keratin fragment

**Figure 5:**
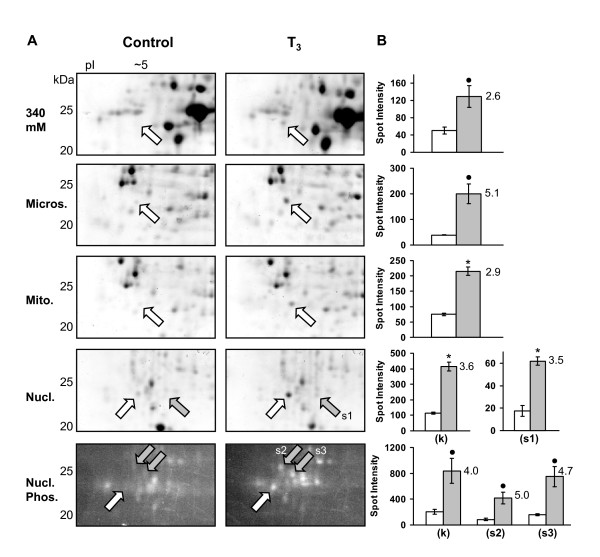
**Identification of a novel *R. catesbeiana *type I (RLK I) keratin fragment by 2D gel analysis**. (A) 2D gel regions of the 340 mM cytosolic, microsomal, mitochondrial, and nuclear fractions show the increase of a protein spot at ~24 kDa and pI ~5 due to T_3 _treatment at 48 h. The corresponding gel region, stained with a phosphoprotein stain, is shown for the nuclear fraction revealing additional changes in the phosphoproteome. The white arrows indicate the spot identified as a novel type I keratin RLK I fragment in the T_3 _samples (see Table 1). In the phosphoprotein gel, the white arrow indicates a possible phosphorylated form of the keratin fragment. The gray arrows indicate an additional unidentified protein and phosphoproteins that are altered upon T_3 _treatment. Relative molecular weights of protein standards are indicated in kDa. (B) Spot density measurements (in arbitrary values) are graphed for the corresponding 2D gels on the left. The white bar represents the control while the gray bar represents the T_3 _treatment. Error bars represent the standard error of the mean from three independent controls and three independent T_3 _samples. Significance is indicated by an asterisk for p < 0.01 and by a black dot for p < 0.04 (ANOVA). The values adjacent to the gray bars represent the fold increase due to T_3_. In the nuclear fraction (k) represents the keratin spot, while (s1) represents an additional protein spot observed to be increased, and (s2) and (s3) represent two phosphoproteins that were increased due to T_3 _treatment. Spot density measurements were normalized between the gels with the β-actin protein spot.

A prominent protein spot at ~24 kDa and pI ~5 was increased upon T_3 _treatment on the 2D gels of several fractions (Fig. 5A). It was observed in the 340 mM cytoplasmic fraction as well as in the microsomal, mitochondrial and nuclear fractions. This protein spot was increased by 2–3 fold as early as 24 h (data not shown), but was more intensely expressed at 48 h (2.6 to 5.1 fold increase depending on the fraction) (Fig. 5B). The greatest increase was observed in the microsomal fraction. The protein spots from each of the fractions were separately analyzed by mass spectrometry proving that each fraction represented the same protein. A combination of electrospray-ionization quadrupole time-of-flight (ESI-QqTOF) and matrix-assisted laser desorption ionization TOF-TOF (MALDI-TOF-TOF) tandem-MS (MS/MS) analyses allowed for peptide sequence information to be obtained for 11 different peptides from this protein (Table 1). Protein database searches with these peptides gave the highest homology match to the *X. laevis *type I keratin 47 kDa protein [NCBI: P05781] also known as *X. laevis *keratin B2 [NCBI: 1304283B) from the XK81 gene family [20].

**Table 1:** MS analysis of protein spot identified to be a type I keratin fragment

**Observed peptide mass (Da, [M+H]^+^)**^1^	**Peptide sequence from MS/MS**^2^	**Identified by MALDI-TOF-TOF**^3^	**% confidence^4^(MS/MS/MALDI)**	**Matched database sequence**^5^
807.4	LAADDFR	Yes	84/89	LAADDFR
809.4	LASYLDK	Yes	100/na	LASYL**E**K
991.5	FENELALR	Yes	100/98	FENELALR
1041.6	LVLQIDNAR	Yes	100/100	**V**VLQIDNA**K**
1073.6	ILAATIDNSR	Yes	100/100	IL**S**ATIDNSR
1079.5	VLDELTMSR	Yes	100/74	VLDELT**LA**R
1184.6	YYDIINDLR	-	96/-	Y**FE**II**S**DLR
1202.6	QSVEADINGLR	-	43/-	QSVE**T**DINGLR
1224.6	NHEEELQVAR	-	73/-	NHEEE**MSI**A**K**
1232.7	-	Yes	-/100	LKFENELALR
1301.6	ALEAANTELELK	-	93/-	ALEAAN**AD**LELK

^1^Observed peptide masses resulting from the tryptic digestion of the protein spot, reported as singly charged. ^2^Peptide sequence information deduced from MS/MS spectra of the corresponding peptides from ESI-QqTOF analysis. The masses of isoleucine are indistinguishable from leucine in MS and therefore L can be I and *vice versa*. ^3^Indicates which peptides were additionally observed with MALDI-TOF-TOF analysis. ^4^Percent confidence for the peptide sequences as reported by PEAKS software for the ESI-QqTOF spectra and by MASCOT for MALDI-TOF-TOF data. ^5^Highest homology match from protein database searching with the observed peptide sequences to *X. laevis *type I keratin 47 kD using SPIDER software. Bold lettering indicates differences between the observed and database sequences.

**Figure 6:**
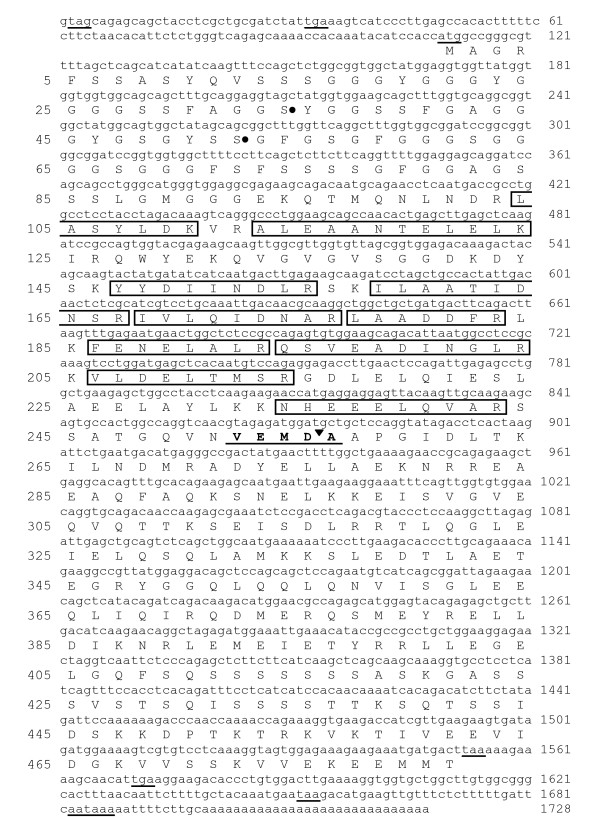
**RLK I cDNA and derived amino acid sequence and location of MS/MS peptide fragments**. The complete nucleotide sequence (lower case) of RLK I cDNA is shown. Underlined nucleotide sequences indicate all the in-frame stop codons, the first methionine codon, and a consensus AATAAA polyadenylation signal. Numbers on the right indicate nucleotide position. Upper case letters indicate the deduced amino acid sequence (single letter code). Boxed sequences indicate tryptic peptides observed in the MS analyses of the RLK I protein spot from the 2D analysis. The underlined VEMDA sequence indicates a consensus caspase cleavage site identified in human type I keratins with the black inverted-triangle indicating the cleavage site. Black dots adjacent to two serine residues indicate possible phosphorylation sites based on those found in human K18 at Ser33 and Ser52 (here Ser34 and Ser52). Numbers on the left indicate amino acid position.

**Figure 7:**
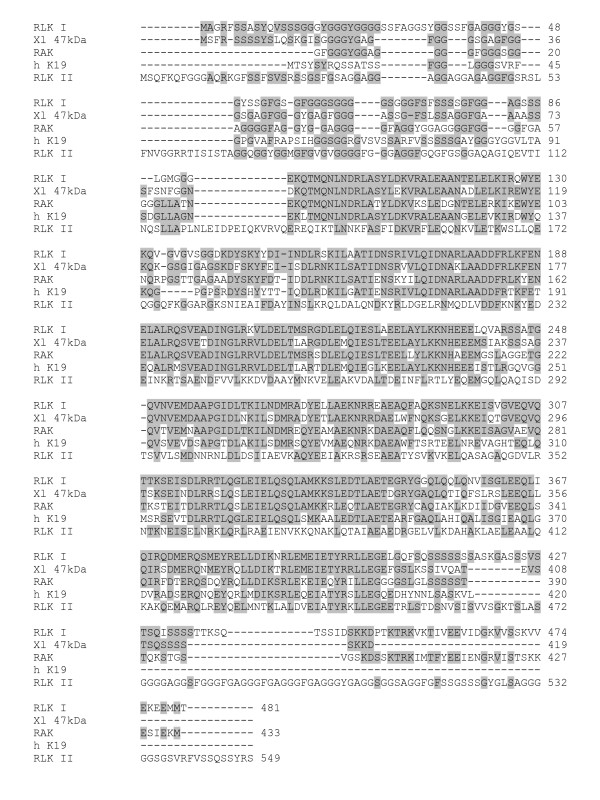
**Multiple sequence alignment of the derived amino acid sequence of RLK I**. The derived amino acid sequence of RLK I [GenBank: EF156435] was aligned with the *X. laevis *type I keratin 47 kDa protein [GenBank: P05781] (Xl 47 kDa), a partial *R. catesbeiana *adult type I keratin RAK [GenBank: BAB47394.1] (RAK), the human type I keratin K19 [GenBank: NP_002267.2] (h K19), and the *R. catesbeiana *larval type II keratin RLK [GenBank: BAB47395] (RLK II) sequences. Gaps that were inserted for optimal alignment are indicated by a dash. Identical amino acids are shaded. Numbers indicate amino acid position for each sequence. The alignment was done using ClustalW software [22].

Parts of the amino acid sequence from two peptides (ALEAANTELELK and NHEEELQVAR) flanking the majority of the peptide sequence identified were used to generate degenerate primers. Degeneracy was limited by taking into account codon usage bias for *R. catesbeiana *and other identified type I keratin cDNA sequences. Two primers with 32 fold degeneracy each generated a single 380 bp PCR product from *R. catesbeiana *tail cDNA. Based on this sequence two gene specific primers (GSP) were designed to perform 5'- and 3'-rapid amplification of cDNA ends (RACE). Two overlapping clones were obtained from the 5'- and 3'-RACE containing the entire open reading frame of this keratin gene (Fig. 6). The cloned sequence was 1728 bp long, with a 109 bp 3'-untranslated region, and a polyadenylation signal, AATAAA, at 17 nucleotides upstream of the poly(A) tract. The deduced amino acid sequence coded for a 481 amino acid protein (predicted size of 52 kDa and pI 5.0) and matched exactly all of the observed peptides from the MS analysis indicating that the correct corresponding cDNA sequence was cloned. BLASTp [21] analysis and ClustalW [22] alignment with this 481 amino acid sequence revealed the highest identity and similarity (80 and 90%, respectively) with the *X. laevis *type I keratin 47 kDa protein [NCBI: P05781] (Fig. 7). About a dozen keratin proteins have been identified for *X. laevis *[23,24]. In *R. catesbeiana *only four keratin proteins have been defined so far. These include the adult and larval keratins, RAK and RLK, respectively, and a keratin K8 and inner-ear cytokeratin [25,26]. RAK is the only acidic type I keratin while the remaining three are basic-neutral type II keratins. The sequence we identified is 73% identical (84% similar) to RAK [NCBI: BAB47394.1] and 32–33% identical to the type II keratins: RLK, *Rana *keratin K8 and inner-ear cytokeratin (Fig. 7 and data not shown). Our sequence also showed 67% identity (81% similarity) to the human type I keratin 19 (K19) protein [NCBI: NP_002267.2]. Based upon this evidence and that presented below, the isolated sequence constitutes a novel *Rana *type I keratin which we will refer to as RLK I. The previous type II larval RLK will be referred to as RLK II.

Keratins are expressed by epithelial cells where they impart a mechanical function. In recent years, they have also been shown to be posttranslationally modified during cell stress, apoptosis, and cell signaling [27,28]. Keratins have been extensively studied in anurans during skin differentiation and metamorphosis. The skin in *R. catesbeiana *transitions from a larval type into a pre-adult type, and finally into an adult type with the onset of metamorphosis [25,26]. These changes are associated with the differentiation and apoptosis of specific epidermal cells and changes in connective tissue [25,29]. Each of these changes have been associated with alterations in keratin type expression in specific cells [23,30]. RLK II expression was reduced in tadpole tail and body skin with the onset of metamorphosis while RAK expression increased [30]. These changes are precociously induced by T_3 _and similar changes have been observed in *X. laevis *with its corresponding larval and adult keratin genes [23,24].

**Figure 8:**
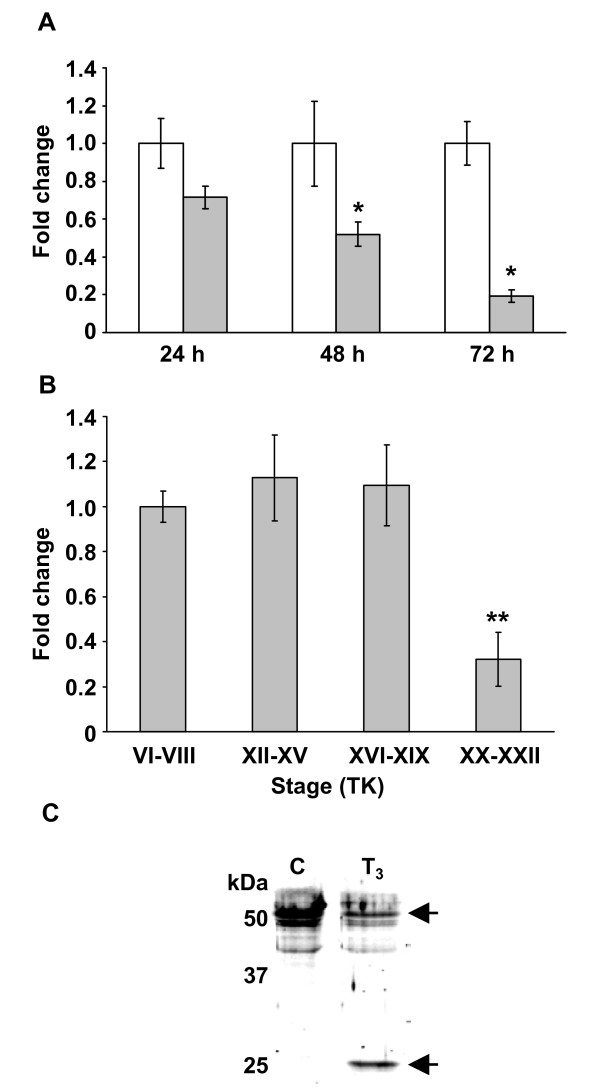
**Changes in transcript and protein fragment levels of RLK I in the tail fin**. (A) Fold change in steady-state levels of the keratin transcript relative to time-matched controls after 100 nM T_3 _exposure for 24, 48 and 72 h. White bars represent controls and gray bars represent T_3 _treatments. Error bars represent the standard error of the mean (n = 4 for all treatments). Significance is indicated by an asterisk for p < 0.03 (Mann-Whitney U). (B) Fold change in steady-state levels of the keratin transcript at different stages of natural metamorphosis relative to premetamorphic TK stage VI-VIII [31]. Error bars represent the standard error of the mean (n = 4 for all treatments). Significance is indicated by a double asterisk for p < 0.002 (Mann-Whitney U). (C) Immunobloting microsomal fraction samples using anti-pan-cytokeratin antibody reveals the appearance of the keratin fragment (25 kDa) and a concomitant loss of keratin at 50 kDa due to 10 nM T_3 _treatment. Relative molecular weights of protein standards are indicated in kDa. Shown is a representative of two independent experiments.

RLK I had the highest homology to the *X. laevis *type I keratin 47 kDa which is expressed during embryonic and larval stages, reduced during metamorphosis, and expressed at very low levels in the adult skin [20]. In order to determine the transcript levels of RLK I, we performed quantitative real-time polymerase chain reaction (QPCR) analysis on tail samples from premetamorphic *R. catesbeiana *tadpoles exposed to T_3 _and during normal tadpole development. Exposure of premetamorphic tadpoles to T_3 _significantly reduced the steady-state levels of the RLK I transcript by 1.9 and 5.2 fold at 48 and 72 h, respectively, relative to time-matched controls (Fig. 8A). The same trend was observed during natural metamorphosis. The steady-state level of the keratin transcript remained unchanged from premetamorphosis [Taylor and Kollros (TK) stage VI-VIII [31] ], through prometamorphosis (TK stage XII-XIX) and then decreased by 3.1 fold upon reaching metamorphic climax (TK stage XX-XXII) when TH levels are maximal and the tail begins to regress (Fig. 8B) [32].

Keratins range in size from 40 to 67 kDa. The keratin spot corresponding to RLK I runs at ~24 kDa and all of the peptides that we detected in the MS analysis from the protein spot mapped to the N-terminal end of the complete cloned sequence (Fig. 6). Immunoblot analyses of the 48 hour microsomal fraction using a pan-cytokeratin antibody revealed the appearance of a similarly migrating fragment in the T_3 _sample with a concomitant reduction of protein intensity at around 50 kDa compared to the control sample (Fig. 8C). Interestingly, the identified peptides lie just upstream of what is known as a consensus caspase cleavage site (VEMDA) identified in type I human keratins [33] suggesting that our observed protein spot is a caspase cleavage product of RLK I (Fig. 6). No caspase cleavage of any keratins has previously been reported in anurans during metamorphosis. However, a number of effector caspases, such as caspase 3 and 7, increase in expression and activity during metamorphosis in the tail [34-36]. Caspase 3 is the most markedly up-regulated in the tail. It is expressed in larval skin epidermal cells that undergo TH-induced cell autonomous death and is known to act on type I keratins in humans [33,37].

RLK I was also highly similar to human K19 protein. This type I keratin and the related K18 protein form heterodimers with the type II keratin K8 in simple-type epithelia and are a prevalent and extensively-studied group of keratins [27,28,38]. Both K18 and K19 are known caspase 3 substrates [33]. Type I keratin caspase cleavage occurs very early in apoptosis before the detection of DNA fragmentation. Type II keratins are not caspase substrates since they lack the caspase cleavage sequence [33]. In addition, the onset of apoptosis is associated with rapid phosphorylation of both type I and type II keratins on their head and tail domains. For K18, Ser52 phosphorylation controls its caspase cleavage and Ser33 phosphorylation regulates binding to 14-3-3 protein [27,33]. From our results it is possible that our keratin fragment is also phosphorylated. Like K18, it contains Ser residues at positions 34 and 52. Also, the phosphoprotein stain revealed an increased phosphoprotein spot in the nuclear fraction positioned just slightly towards the more acidic end of the gel relative to the RLK I fragment. This phosphoprotein was increased at 24 and 48 h by 1.6 and 4.0 fold respectively which matches the increase of the total-protein keratin spot (Fig. 5A and 5B). Although this phosphoprotein could not be identified by MS, its more acidic position adjacent to the RLK I fragment suggests it could be a phosphorylated form of the same fragment.

The reasons for keratin cleavage and phosphorylation are not well understood and are speculated to function as phosphate sinks, for filament re-organization during apoptosis, or mechanisms that protect cells from apoptotic damage allowing a graded sequence of events. In human cancer therapy, the appearance of the caspase cleavage products of K18 and K19 in patient serum are used as indicators of cancer prognosis and apoptotic death of tumor cells undergoing chemotherapy [39,40]. The ease with which this fragment was detected in our immunoblot using a pan-cytokeratin-specific antibody suggests that it could be used as a protein marker for the induction of a TH response in tadpoles which may be perturbed upon exposure to disruptors of TH action. The very early appearance of this fragment during precocious tail metamorphosis before the detection of any overt apoptotic events [9] is very intriguing. It is currently unclear whether the appearance of this keratin fragment is a product of initiation of the apoptotic event or is required for tail regression to occur.

### Phosphorylation changes in γ-interferon-inducible lysosomal thiol reductase

**Figure 9:**
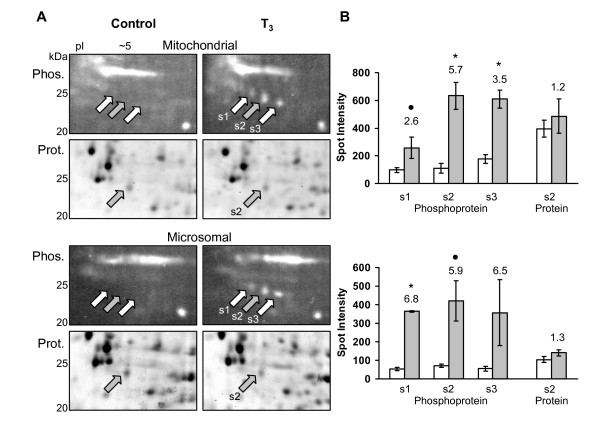
**Phosphorylation changes in γ-interferon-inducible lysosomal thiol reductase (GILT)**. (A) Phosphoprotein 2D gel regions of the mitochondrial and microsomal fractions showing the increase in a row of phosphoprotein spots (s1, s2, s3) due to T_3 _treatment at 48 h, while a corresponding total-protein stained 2D region shows no change in the only detectable protein spot s2 (gray arrows). Relative molecular weights of protein standards are indicated in kDa. (B) Spot density measurements (in arbitrary values) are graphed for the corresponding 2D gels on the left. The white bar represents the control while the gray bar represents the T_3 _treatment. Error bars represent the standard error of the mean from three independent controls and three independent T_3 _samples. Significance is indicated by an asterisk for p < 0.01 and by black dot for p < 0.1 (ANOVA). The values adjacent to the gray bars represent the fold increase due to T_3_. Phosphoprotein spots s1, s2, and s3 increase while the corresponding protein spot s2 does not change. MS analysis of the only detectable protein spot s2 (gray arrow) is indicated in table 2. Spot density measurements were normalized between the gels with the β-actin protein spot.

Three phosphoprotein spots located at 25 kDa and pI ~5 increased in abundance due to T_3 _at 48 h in the mitochondrial and microsomal protein fractions (Fig. 9A). These spots formed a train indicating that this could be a single protein with different posttranslational modifications. The phosphoprotein spots increased ~5 fold, while the only corresponding protein spot that could be detected on the total-protein gels did not change in abundance (Fig. 9B). This indicates that T_3 _caused a change in the posttranslational state of the protein while its expression level remained the same. MS analysis of the protein spot from the microsomal and mitochondrial, control and treatment samples indicated that this is the same protein spot (data not shown). ESI-QqTOF MS/MS analysis of the tryptic fragments of the protein spot provided two high quality peptide sequences (Table 2). A homology search of the protein database with the two sequences gave the highest match to the *Xenopus tropicalis *γ-interferon-inducible protein 30 (IP30) [NCBI: NP_001017196.1], more commonly known as gamma-interferon-inducible lysosomal thiol reductase (GILT) [41]. This protein also matched the observed molecular weight and pI point.

**Table 2:** MS analysis of protein spot identified as GILT

**Observed peptide mass (Da, [M+H]^+^)**^1^	**Peptide sequence from MS/MS**^2^	**% confidence**^3^	**Matched database sequence**^4^	**E value**^5^
1399.8	(CL)FNLVTELYK	100 (98)	Observed 1 CLFNLVTELYK 11 LFNLV + YK	1877
			Database 223 SLFNLVCDTYK 233	

1405.7	(TV)LDCVDGDLGNK	100 (90)	Observed 1 TVLDCVDGDLGNK 13 TVL+CV+GDLGNK	0.001
			Database 172 TVLECVNGDLGNK 184	

MS analysis of the only detectable protein spot s2 (gray arrows in Fig. 9). ^1^Observed peptide masses resulting from the tryptic digestion of the protein spot s2, reported as singly charged. ^2^Peptide sequence information deduced from MS/MS spectra of the corresponding peptides from ESI-QqTOF analysis. The masses of isoleucine are indistinguishable from leucine in MS and therefore L can be I and *vice versa*. ^3^Percent confidence for the peptide sequences, as reported by PEAKS software for the ESI-QqTOF spectra. ^4^Highest homology match from protein database searching with the observed peptide sequences to *X. tropicalis *GILT. Indicates the sequence alignment of the observed peptide to the identified protein (Database) as aligned by BLASTp. The sequence in between indicates the matching and similar (+) amino acids between the two sequences.^5^From BLASTp alignments.

GILT is the specific lysosomal enzyme responsible for thiol reduction of proteins in the endocytic pathway for antigen presentation [41]. Gamma interferon (IFNγ) released by activated T cells, increases antigen presentation on antigen presenting cells (APCs) such as macrophages, dendritic cells, and B cells through the induction of major histocompatability complex II (MHCII) molecules and related proteins. In addition, IFNγ increases the constitutive expression of GILT in APCs and even induces GILT expression in non-hematopoietic cells such as fibroblasts, keratinocytes and endothelial cells [42]. GILT is transported in its proform (30–35 kDa) from the ER and Golgi complex into the early endosomes of the endocytic pathway where it is combined with MHCII-invariant chain containing vesicles and converted into its mature form (25–30 kDa) by the removal of N- and C-terminal prosequences in the late endosomes and lysosomes [41,43]. It is likely that the GILT protein we identified is the mature form of the protein due to its localization in the microsomal and mitochondrial fractions (which contain vesicles from the ER, Golgi and lysosomes), the size on the 2D gels, and scope of coverage of identified peptides.

Further analyses of the phosphoprotein spots using 2D immunoblots and antibodies specific for the three common phospho-amino acids, phosphoserine, phosphothreonine, and phosphotyrosine did not give any indication that the GILT protein is differentially phosphorylated on any of those residues (data not shown), while the ProQ Diamond phosphoprotein stain reproducibly detected a change in phosphorylation. As with many lysosomal enzymes, GILT is a glycoprotein with three potential N-linked glycosylation sites. Furthermore, mature GILT contains mannose-6-phosphate (M6P) on one or more of the glycan chains [41]. Phosphorylation of mannose within the N-linked glycan chain is a signal recognized in the Golgi complex by the M6P receptor that targets lysosomal enzymes to the endocytic pathway [44]. Therefore, it is possible that the ProQ Diamond phosphoprotein stain is detecting a phosphate in M6P and that our observed increase in phosphorylation of GILT indicates an increased amount of M6P-containing GILT being directed into the endocytic pathway due to T_3_. In accordance with this idea of increased endocytic pathway activity, the lysosomal endoprotease, cathepsin D, increases in expression due to T_3 _in our iTRAQ experiment (see below). Cathepsin D, with other lysosomal cathepsin proteases (B, L and S) also converts the proform of GILT into its mature form by cleaving off the N- and C-terminal prosequences [43,45]. A GILT knock-out study in mice, showed that GILT is required for the presentation of disulfide-containing antigens and the resulting T cell activation [46]. These ideas, and the additional observation of increased immunoglobulin (Ig) heavy chains from our iTRAQ experiment (see below), indicate that T_3 _must be stimulating antigen presentation and possibly in turn activating T cell and finally Ig-producing/possessing B cell lymphocytes.

### Additional changes observed in the 2D gel analysis

Within the region of the keratin spot, additional proteins and phosphoproteins were increased in the nuclear fraction (Fig. 5A). A protein spot that could not be identified increased by 2 (data not shown) and 3.5 fold at 24 and 48 h, respectively (Fig. 5B). And two unidentified phosphoproteins were increased only at 48 h by ~5 fold (Fig. 5A and 5B). A protein spot located at 30 kDa and pI ~5.5 in the microsomal fraction was increased by 2.4 fold at 48 h upon T_3 _exposure [see Additional file 1]. Amino acid sequence was obtained with high confidence for three peptides from this spot but no significant homology match could be made [see Additional file 2].

### Differential expression analysis using iTRAQ

2D gel analysis has the advantage of identifying specific protein isoforms and posttranslational modifications of whole proteins. This whole-protein analysis approach is limited by the inability to observe hydrophobic proteins and proteins with extremes in size or pI [47]. In contrast, MS analysis techniques circumvent this limit by analyzing peptides derived from trypsin-cleaved protein samples. However MS peptide analysis has limitations in distinguishing between protein isoforms that potentially share identical peptides [48]. Consequently, 2D gel analysis and MS analysis techniques are complementary methods that can be combined to study differential protein expression. Therefore, to increase the number of observed altered proteins, changes in protein expression in the *R. catesbeiana *tadpole tail fin were additionally assessed using the novel MS technique, iTRAQ [49].

**Figure 10:**
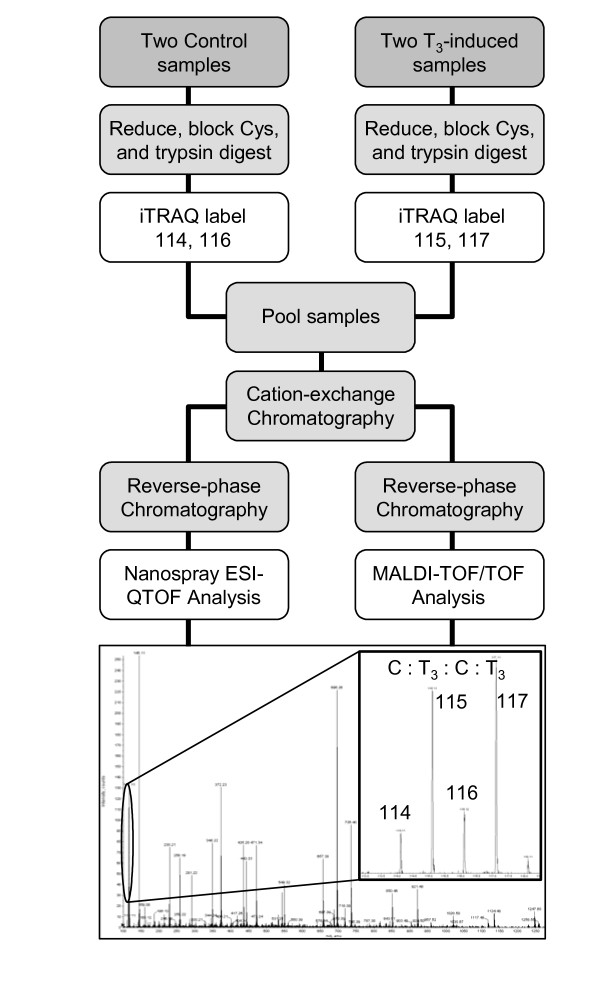
**iTRAQ analysis**. Two control and two treatment samples were each labeled with one of the four iTRAQ tags as shown. The peptide samples were pooled, fractionated by two dimensions of liquid chromatography (cation-exchange and reverse-phase), and analyzed by MS. The iTRAQ sample was analyzed three times on an ESI-QqTOF mass spectrometer and once on a MALDI-TOF-TOF mass spectrometer. The image shows a sample MS/MS spectrum of a single peptide from which the amino acid sequence is deduced, and in addition, it reveals the four reporter ions (enlarged region) from the iTRAQ tags, in the low-mass region, whose intensity indicates the relative abundance of that peptide in the four samples. The two controls are labeled with tags 114 and 116, and show reporter ions at that m/z, while the two T_3 _treatment samples are labeled with tags 115 and 117, showing reporters at those m/z. This spectrum reveals an increase in that peptide due to T_3_.

The iTRAQ labeling reagents are four unique chemical tags (114, 115, 116 and 117) that label peptides on primary amines allowing for the quantitation of relative protein abundance in four samples simultaneously during a single analysis (Fig. 10). Each sample is labeled with a different tag. The tags are isobaric, meaning that identical peptides from four different samples will be observed in the MS analysis as a single peptide. Peptide fragmentation and tandem-MS (MS/MS) analysis of that peptide then allows peptide sequence information to be acquired leading to a protein inference. In addition, different reporter ions are generated from the tags after peptide fragmentation at 114, 115, 116 and 117 m/z in the MS/MS spectra, representing each of the four samples, indicating the proteins' relative abundance. Relative abundance is obtained by measuring the area underneath the reporter peaks and is reported as a ratio between the samples. In our case, the data is reported as the average ratio of the T_3 _treatment *versus *the control from two duplicate experiments. A ratio above 1 indicates the fold increase in expression due to T_3 _exposure, while a ratio below 1 indicates a reciprocal fold decrease in expression.

Traditionally, iTRAQ data analysis is performed automatically with software, which greatly reduces the analysis by the end-user. The success of obtaining valid data in this manner, however, is first dependent on the protein identification obtained using software, such as MASCOT, where an identification is reliant on a perfect match of ion peaks in a MS/MS spectrum to an existing protein in the protein sequence database [50]. This method works well for the analysis of organisms with extensive entries in gene and protein databases but results in many unidentified spectra when less well-defined species, such as *R. catesbeiana*, are analyzed. Additionally, when a high quality partial MS/MS spectrum is obtained (yielding partial peptide sequence information) software limitations do not permit identification when using ion peaks database searching.

To remedy these problems, we first analyzed the raw iTRAQ data using Excel spreadsheets to find all the MS/MS spectra that showed significant changes in expression due to the T_3 _treatment. The resulting MS/MS spectra were *de novo *sequenced manually and/or using additional software (PEAKS) [51]. The resulting peptide sequences were then used to query protein databases using BLASTp. This resulted in substantially higher identification of proteins due to homologous matches or identical matches originally missed by MASCOT.

To increase the proteome coverage and quantitation accuracy, the iTRAQ samples were analyzed three times on an ESI-QqTOF mass spectrometer (Fig. 10) [52]. As shown in Table 3, many of the peptides that showed changes in abundance due to T_3 _treatment overlapped between the runs. The mass spectrometer collected 6249 to 7682 unique MS/MS spectra per run. Out of those, ~0.3% of the spectra with high quality iTRAQ reporter tags represented peptides that changed according to our criteria of 1.5 fold or higher. In total, 41 unique spectra showed an increase or decrease of 1.5 fold or higher due to the T_3 _treatment. Successful *de novo *peptide sequence information was obtained for 34 (83%) of those spectra. Nineteen (56%) of those amino acid sequences were identified with homology protein database searching. Only three of the 19 peptides were identified using the standard database matching method that requires a perfect match to a protein database using MASCOT software. These 19 peptides represent at least 11 different proteins, 5 of which increased and 6 of which decreased in abundance due to T_3 _exposure.

**Table 3:** Summary of results for iTRAQ analysis by ESI-QqTOF

	**Run 1**	**Run 2**	**Run 3**	**Unique**^1^
**Unique spectra**^2^	7682	6249	6361	n/a
**Changing peptides**^3^	17	17	21	**41**
***de novo *sequenced**^4^	13	16	18	**34**
**Homology matched**^5^	8	7	11	**19**
**MASCOT ID**^6^	1	1	2	**3**

**Total proteins ID**^7^	**11 change:**	**5 up**	**6 down**	

The iTRAQ samples were analyzed three times on an ESI-QqTOF mass spectrometer. ^1^Indicates number of unique spectra shared between the replicate runs. ^2^Total number of unique spectra recorded per MS analysis run. These overlap by unknown amount: n/a. ^3^Number of spectra that change by ≥ 1.5 fold due to T_3_, have good quality reporter tags, and a ratio of the two controls between 0.67 and 1.5. ^4^Number of spectra for which amino acid sequence could be obtained through manual sequencing. ^5^Number of spectra for which a protein inference was made with BLASTp. ^6^Indicates how many spectra were identified using the standard iTRAQ analysis with MASCOT software. ^7^Number of different proteins identified from the resulting peptides and their change in abundance.

The same iTRAQ samples were also analyzed using a MALDI-TOF-TOF mass spectrometer (Fig. 10) to obtain additional proteome coverage because the peptide ionization characteristics are different from the ESI-QqTOF. Due to software limitations no *de novo *sequencing of raw MS/MS spectra was performed and the data was analyzed using the standard method of database searching using the observed MS/MS spectra ion peaks using MASCOT. The MALDI-TOF-TOF analysis identified 3352 peptides with over 95% confidence. These peptides represented 729 unique proteins, of which 50% were identified through a single peptide. Four proteins showed altered abundance ≥ 1.5 fold due to the T_3 _treatment. One of these proteins had an increased abundance while three had a decreased abundance. Details of the iTRAQ results are presented in Table 4. Interestingly, some of these proteins have established functional connections that may be important during tail regression. In addition, some of the proteins have connections to those identified in the previous 2D gel analyses. The identified proteins are involved in apoptotic events, modulation of the extracellular matrix, immune system, metabolism, mechanical function, and oxygen transport and are discussed in detail below.

**Table 4:** Differentially expressed proteins in the *Rana catesbeiana *tail fin due to T_3_-induction as analyzed by iTRAQ

**Protein name [accession #/Species]**^1^	**Fold change**^2^	**Observed peptide mass (Da, iTRAQ [M+H]^+^)**^3^	**Observed peptide sequence**^4^	**% Confidence**^5^	**Matched database sequence**^6^	**E value**^7^
**INCREASED**						
MGC80395 protein (Sterol regulatory element-binding transcription factor 2) [AAH72922/*Xenopus laevis*]	1.6	2949.6	LTPATVET (frag.)	manual	Query 1 LTPATVET 8 LTPATV+T Sbjct 184 LTPATVQT 191	136
Inter-α inhibitor H4 [XP_848765/*Canis familiaris*]	2.1	1789.1	V(TFE)LVYEEMLK	90–100 (53)	Query 1 VTFELVYEEMLK 12 VTFELVYEE+LK Sbjct 140 VTFELVYEELLK 151	0.004
Hemoglobin α-III chain, larval [P02011/*Rana catesbeiana*]	1.5	1224.7	FLSFPQTK (frag.)	manual	Query 1 FLSFPQTK 8 FLSFPQTK Sbjct 33 FLSFPQTK 40	5.4
Hemoglobin α-III chain, larval [P02011/*Rana catesbeiana*]	1.5	1250.8	FLSFPQTK (frag.)	100	Query 1 FLSFPQTK 8 FLSFPQTK Sbjct 33 FLSFPQTK 40	5.4
Hemoglobin α-III chain, larval [P02011/*Rana catesbeiana*]	1.5	2067.0	YVPHFDLTPGSADLNK	99	Query 1 YVPHFDLTPGSADLN 15 Y PHFDLTPGSADLN Sbjct 42 YFPHFDLTPGSADLN 56	9 e-05
MGC80107 protein (Biliverdin reductase B) [AAH72790/*Xenopus laevis*]	1.5	1904.3	VISTPDLSHFFLR	100	Query 1 VISTPDLSHFFLR 13 VIST DLS FFLR Sbjct 174 VISTHDLSLFFLR 186	0.26
Immunoglobulin heavy chain [AAC12909/*Hydrolagus colliei*]	1.6	1237.8	VVLLPPSPK	99	Query 1 VVLLPPSP 8 V+LLPPSP Sbjct 133 VILLPPSP 140	63
Immunoglobulin heavy prechain [CAA33212/*Xenopus laevis*]	2.0	1817.9	SDPDQGFDGTYTVK	manual	Query 1 SDPDQGFDGTYTVK 14 S P++ +DGT+TVK Sbjct 393 SAPEKAYDGTFTVK 406	52
Immunoglobulin heavy chain constant region [AAC12914/*Hydrolagus colliei*]	46	1391.7	LNVADWNSGK	99	Query 1 LNVA--DWNSGK 10 LNV+ DW SGK Sbjct 69 LNVSTEDWKSGK 80	170
Immunoglobulin M heavy chain [AAO37747/*Ornithorhynchus anatinus*]	1.5	2065.0	FTCTVSHSDLPAPVEK	95	Query 1 FTCTVSHSDLPAP 13 FTCTVSH+DLPAP Sbjct 446 FTCTVSHADLPAP 458	7 e-04
Immunoglobulin heavy chain variable region [AAP41191/*Lepus granatensis*]	1.6	1930.3	(RKQ)VVEAGGALIK	100 (92–96)	Query 3 QVVEEAGGALIK 14 Q VEE+GG LIK Sbjct 1 SAPEKAYDGTFTVK 12	22
Immunoglobulin heavy prechain [CAA33212/*Xenopus laevis*]	1.6	1471.8	DQGFDGTYTVK	manual	Query 4 FDGTYTVK 11 +DGT+TVK Sbjct 399 YDGTFTVK 406	9.2
LOC443721 protein (cathepsin D) [AAH94178/*Xenopus laevis*]	1.7	980.5	AYWQIR	98 (MALDI)	Query 1 AYWQIR 6 AYWQIR Sbjct 257 AYWQIR 262	42
**DECREASED**						
α-2-macroglobulin [AAY98517/*Xenopus laevis*]	0.67	2195.2	AYVTV(LGD)IMGTALENLDR	97–100 (41)	Query 1 AYVTVLGDIMGTALENLDR 19 AYVTVLGDIMGTA++NLDR Sbjct 958 AYVTVLGDIMGTAMQNLDR 976	1 E-08
Calcium-binding protein p26olf [BAA34388/*Rana catesbeiana*]	0.47	1312.7	GNTTSMNFK	manual	Query 1 GNTTSMNFK 9 GNTTSMNFK Sbjct 31 GNTTSMNFK 39	0.57
α1type I collagen [BAA29028/*Rana catesbeiana*]	0.52	2111.2	TGPAGAPGQDGRPGPPGPPGAR	manual	Query 1 TGPAGAPGQDGRPGPPGPPGAR 22 TGPAGAPGQDGRPGPPGPPGAR Sbjct 538 TGPAGAPGQDGRPGPPGPPGAR 559	6 e-12
α1type I collagen [BAA29028/*Rana catesbeiana*]	0.52	2418.2	PPGPSGEK (frag.)	97	Query 1 PPGPSGEK 8 PPGPSGEK Sbjct 912 PPGPSGEK 919	17
Caridac α actin 2 [AAX85445/*Rana catesbeiana*]	0.38	2244.3	VAPEEH(PT)LLTEAPLNPK	93–100 (66)	Query 1 VAPEEHPTLLTEAPLNPK 18 VAPEEHPTLLTEAPLNPK Sbjct 98 VAPEEHPTLLTEAPLNPK 115	3 e-09
myosin heavy chain (skeletal muscle MHC-3) [AAD13771/*Rana catesbeiana*]	0.39	2491.9	FQAALEEAEASLEHEEAK	manual	Query 1 FQAALEEAEASLEHEEAK 18 FQAALEEAEASLEHEEAK Sbjct 320 FQAALEEAEASLEHEEAK 337	8 e-09
Mylpf-prov protein (myosin light chain 2) [AAH41503/*Xenopus laevis*]	0.38	2098.2	NICYVITHGED (frag.)	100	Query 1 NICYVITHGED 11 NICYVITHGED Sbjct 156 NICYVITHGED 166	0.002
MGC68533 protein (coatomer protein complex, subunit γ) [AAH61661/*Xenopus laevis*]	0.61	1491.6	NAHSLYLAGVFR	99 (MALDI)	Query 1 NAHSLYLAGVFR 12 NAHSLYLAGVFR Sbjct 824 NAHSLYLAGVFR 835	0.002
Cortactin [NP_005222/*Homo sapiens*]	0.65	1585.9	YGLFPANYVELR	98 (MALDI)	Query 1 YGLFPANYVELR 12 YLFPANYVELR Sbjct 538 YGLFPANYVELR 549	4 e-04
Triose phosphate isomerase [NP_788764/*Drosophila melanogaster*]	0.64	1711.8	DIGADWVILGHSER	100 (MALDI)	Query 1 DIGADWVILGHSER 14 DIGADWVILGHSER Sbjct 185 DIGADWVILGHSER 198	7 e-06

^1^Name of protein for the highest scoring match that resulted with the observed peptide sequence using BLASTp (2.2.15) and the NCBI protein database (Oct. 15, 2006) searching all metazoans. The NCBI accession number and the species name for the identified protein are shown. ^2^Fold change ratios greater then 1.0 indicate a fold increase, and those below 1.0 indicate the reciprocal of fold decrease. The number is the average fold change of two replicate experiments, which consisted of two treatment samples and two control samples. ^3^Observed mass of the peptide in the MS analysis, modified with the iTRAQ reagent, reported as singly charged. ^4^Amino acid sequence determined for the observed peptide by manual *de novo *or automatic *de novo *by PEAKS software sequencing, or a MASCOT software match. (frag.) indicates that only a partial peptide sequence could be determined. The masses of isoleucine are indistinguishable from leucine in MS and therefore L can be I and *vice versa*. ^5^Percent confidence for the peptide sequence as reported by the PEAKS or MASCOT (MALDI) software. (MALDI) indicates that the peptide was identified in the MALDI-TOF-TOF analysis of iTRAQ samples. "manual" indicates that the peptide sequence was obtained manually and no percent confidence can be reported. ^6^Indicates the sequence alignment of the observed peptide (Query) to the identified protein (Sbjct) as aligned by BLASTp. The sequence in between indicates the matching and similar (+) amino acids between the two sequences. ^7^From BLASTp alignments.

### Immune system components

T_3 _induces the activation of the immune system as is evident with the increased maturation of GILT as revealed in the 2D analysis. In addition, this is apparent from an increase in immunoglobulin chains and cathepsin D and is possibly related to the decrease of a coatomer protein.

Six peptides that increased 1.5–4.6 fold matched a type of immunoglobulin heavy chain (Table 4). The peptides matched sequences from *X. laevis *as well as from other species. The peptides were derived from constant and variable regions of the heavy chain, and one matched the IgM heavy chain isotype.

The immune system plays a major role during the metamorphic process. Macrophages increase in the regressing tadpole tail in number and phagocytic activity removing apoptotic bodies of dying muscle cells [53]. Larval lymphocyte populations rise in the growing tadpole, decrease sharply at climax of metamorphosis, and expand again with adult lymphocytes [54]. The exchange in lymphocyte populations appears to be required for the tolerance of new adult antigens and for the removal of larval tissues that are seen as 'non-self' in the metamorphosing animal. *X. laevis *tail cells possess larval antigens that stimulate the immune system of a syngeneic adult or a metamorphosing animal [55]. Izutsu and coworkers have shown that a 59 kDa larval antigen expressed specifically by tail epidermal cells only during metamorphosis causes increased proliferation of newly arising adult-type T lymphocytes, eventually leading to the destruction of tail fin tissue by cytotoxic T lymphocytes (CTL) and natural killer cells (NK) [56,57]. Collaboration between T- and B-cell types must occur for NK cell activation and may explain our observation of increased immunoglobulin heavy chains which can only come from B cell activation. A reported peptide sequence from the 59 kDa antigen is homologous to keratin α, and points at the possibility that the increased keratin fragment observed in the 2D gel analyses may be a larval antigen produced to direct the involvement of the immune system.

A peptide that increased by 1.7 fold was identified in the MALDI-TOF-TOF analysis corresponding to a putative *Xenopus *cathepsin D protein (Table 4). Cathepsin D is a lysosomal aspartyl protease that has been previously implicated in tadpole tail regression [58,59]. The increase in cathepsin D may be related to GILT maturation inside the lysosomes and a resulting increase in antigen presentation as discussed above.

A subunit γ protein of the coatomer protein complex (COPI) corresponding to the *X. laevis *MGC68533 protein decreased by 1.6 fold in the MALDI-TOF-TOF analysis (Table 4). Protein transport between the ER and Golgi compartment is mediated by these non-clathrin-coated vesicular coat protein complexes [60]. COPI is composed of seven unique subunits and coats the vesicles on the cytoplasmic side as they are transported from one organelle to another. COPI is mainly associated with retrograde transport of vesicles from the Golgi back to the ER to retrieve escaped ER-resident proteins and vesicle machinery. The γ subunit binds double lysine motifs in the transmembrane proteins targeted for return to the ER. Interestingly, GILT and other lysosomal zymogens have dilysine motifs which are used as cleavage sites during enzyme maturation. There have not been any previous associations of COPI with TH or metamorphosis, but it is possible that T_3 _causes a specific change in vesicle trafficking to accomodate tail regression. This change may contribute to an increase in mature GILT in the lysosomes as is observed on the 2Ds of the microsomal and mitochondrial fractions.

### Extracellular matrix components and modifier proteins

Changes in the extracellular matrix (ECM) of the regressing tadpole tail have been well documented [1]. Four proteins involved in ECM structure and modification were identified by iTRAQ (Table 4).

The plasma proteinase inhibitor, α-2-macroglobulin (A2M), was decreased 1.5 fold upon T_3 _treatment (Table 4). A2M is synthesized by several cell types such as hepatocytes, lung fibroblasts and monocyte-macrophages, and functions to trap and facilitate clearing of proteases such as trypsin, thrombin and collagenases [61,62]. Tissue-specific A2M synthesis is independent of plasma A2M and serves a compartmentalized function such as the regulation of tissue proteinases such as matrix metalloproteinases. Since there is an increase in matrix metalloprotease activity in the tadpole tail in TH-induced and natural metamorphosis [1], the observed decrease in the A2M levels could be a result of an organized downregulation to allow for ECM remodeling or A2M and metalloprotease covalent linkage and subsequent removal and degradation of the proteins. Recently, Lin *et al. *observed a T_3_-dependent decrease in A2M transcripts in a human hepatocellular carcinoma cell line [63].

A peptide matching inter-α inhibitor H4 protein increased by 2.1 fold (Table 4). The H4 polypeptide is a less described component of the trimeric plasma serine protease inhibitor, inter-α inhibitor (IaI), more commonly composed of the H1, H2 and bikunin polypeptides [64,65]. IaI proteins are linked to inflammation processes and ECM structure. Serine protease inhibitor activity of bikunin regulates plasmin which is a known activator of matrix metalloproteases [64]. The H4 polypeptide is also a serum marker for the acute-phase response in infected animals and humans resulting from the nonspecific immune response [66-68] and may be an additional link to our observation of the immune system activation. Additionally, the H polypeptides stabilize and protect the ECM through covalent links to ECM components and possibly through collagen binding domains [64].

α 1 type I collagen (α 1(I) chain) was identified by two peptides that decreased by 1.6–1.9 fold (Table 4). Type I collagen is the most abundant member of the collagen family and is the main constituent of the ECM [69]. It is a major substrate of matrix metalloproteinase 1 (MMP1) which is upregulated in *R. catesbeiana *tail and back skin during natural and T_3_-induced metamorphosis [70,71]. Collagen (α 1(I) and α 2(I)) gene expression also decreased under these conditions in the tail and intestine [72-74]. It is possible that the α 1(I) collagen gene is negatively regulated by a TRE-like site as in rat cardiac fibroblasts [75]. Here we show that this change also occurs at the protein level.

The last peptide associated with ECM remodeling proteins corresponds with cortactin (Table 4). A 1.5 fold decrease in this Src homology 3 (SH3) domain-containing protein suggests altered tyrosine kinase signaling of actin cytoskeleton rearrangements [76]. Cortactin is a filamentous-actin binding protein that stabilizes actin networks and promotes actin polymerization. Since cortactin interacts with various proteins involved in actin polymerization, endocytosis (recall an overlap with GILT and cathepsin D proteins) and cell-cell interactions, modification of any or all of these processes could contribute to tail regression.

### Cell metabolism proteins

Two proteins involved in cell metabolic homeostasis were altered in abundance by T_3 _treatment (Table 4). The first peptide was increased by 1.6 fold matched with a sterol regulatory element binding protein-2 (SREBP-2). This membrane-bound transcription factor regulates lipid homeostasis through the control of genes encoding key enzymes important in cholesterol synthesis [77]. The SREBP transcription factors have not been implicated in anuran metamorphosis before, however, they are involved with TH in the mammalian context [78-80]. Recently Shin and Osborne [78] observed that T_3 _regulates both SREBP-2 mRNA and nuclear protein levels, as well as SREBP-2-controlled genes such as the low density lipoprotein (LDL) receptor in mice. The SREBP-2 promoter contains at least one TR-binding site and is activated directly by TR in a ligand-dependent manner [78]. It is therefore possible that the increased level of SREBP-2 observed in our experiments may be a direct response to T_3_. This remains to be determined.

The second peptide was reduced by 1.6 fold and corresponds to triose phosphate isomerase (TIM; Table 4). This enzyme is involved in glycolysis [81] and its decrease is compatible with a shift from glycolysis to oxidative phosphorylation observed during metamorphosis [82].

In addition, two proteins involved in oxygen transport were increased upon T_3 _treatment. Three peptides corresponding to larval hemoglobin α-III chain increased by 1.5 fold (Table 4). This protein is only found in tadpole red blood cells (RBCs) and not adult RBCs [83,84]. In *R. catesbeiana *there is a change in erythropoiesis sites and hemoglobin types during tadpole growth and metamorphosis that is influenced by THs [83,85-88]. Young tadpoles produce hemoglobin components I and II in the kidneys, older tadpoles produce components III and IV in the liver, and after metamorphosis, only adult hemoglobins are produced in the bone marrow. This sequential change produces hemoglobins with lower oxygen affinities. Our observation of an increase in hemoglobin α-III is indicative of an accelerated progression of hemoglobin switching due to T_3 _exposure.

Consistent with a switch in hemoglobin is an 1.5 fold increase in a peptide matching biliverdin reductase B (BVR-B; Table 4). This enzyme is important in hemoglobin metabolism and is predominantly found in human fetal liver [89]. There, it produces bilirubin IXβ from biliverdin derived from heme. Bilirubin IXβ is the major heme catabolite during human fetal development [90]. In adult human liver biliverdin reductase A (BVR-A) is the major form and it produces bilirubin IXα. The switch in this heme degradation pathway seems to be related to the switch in hemoglobins from fetal to adult forms.

### Mechanical function and calcium modulated proteins

The abundance of peptides from muscle α-actin, myosin heavy chain (MHC) and myosin light chain (MLC) was reduced by exactly the same amount (2.6 fold; Table 4). These proteins are the fundamental components of the molecular machinery used in muscle contraction. The peptide for muscle α-actin is distinguished from the β and γ forms of cytoplasmic actins by a T residue instead of V in the HPTLL sequence within this peptide. The peptide sequence matches the *R. catesbeiana *cardiac α-actin 2 sequence perfectly as well as other muscle α actins in other species. The MHC peptide matches the *R. catesbeiana *skeletal muscle MHC-3 and is distinguishable from the other isoforms, MHCs -1, 2, 4, and 5, by three residues in this species [91]. The MLC peptide matches the *X. laevis *MLC-2 (Mylpf-prov protein) as well as MLC-2 proteins from other species.

An embryonic/larval α-3 skeletal-actin gene has been found in *Xenopus borealis *[92]. This gene was expressed in the skeletal muscle of embryos and tadpoles, but not in adults. However, the corresponding gene in *X. laevis *is highly expressed in both adult muscle and at earlier stages. The human skeletal muscle α-actin gene is a direct TH response gene in cardiocytes [93,94]. The cardiac α-actin gene of rats is also inducible with T_3 _in heart myocytes [95].

Five skeletal muscle MHC isoforms have been cloned in *R. catesbeiana *[91]. No specific larval forms have been found and all five isoforms are expressed in the tail and limb muscle of both tadpoles and adults. In the tadpole tail muscle, T_3 _increases the mRNA for MHCs -1, 3, 4, and 5 by two to three-fold within 48 h [91]. However, in contrast, the rate of total MHC protein synthesis was reduced by 40% at 48 h [91] consistent with our observations for MHC-3. In fast twitch muscle fibers, MHC-3 is associated with MLC-2 [96]. MLCs are Ca^2+^-signaling dependent regulators of muscle contraction and have been investigated in *X. laevis *and *R. pipiens *but their responses to TH are not well understood [96]. Our data suggest that MHC and MLC expression are co-regulated.

A peptide that was decreased by 2.1 fold matched the p26olf protein of *R. catesbeiana *(Table 4) The p26olf protein is a S100-like Ca^2+^-binding protein with the very unique feature of having two S100-like regions [97]. Thus far p26olf has only been found in the anurans *R. catesbeiana, X. tropicalis *and *X. laevis*, and appears not to have any homologues in other vertebrates. Immunohistochemical studies on *R. catesbeiana *frogs showed that p26olf primarily localizes in the cilia of olfactory and lung respiratory epithelia. p26olf may be involved in olfactory signal transduction and a related protein, S100/A11 (calgizzarin), is a key mediator of high Ca^2+^-induced growth arrest and differentiation in human keratinocytes [98]. In addition, Makino *et al. *[99] have shown that introducing an N-terminal peptide of S100/A11 into human carcinoma cell lines induced apoptosis. Thus, it may be possible that p26olf is involved in the control of apoptotic events in the tadpole tail.

## Conclusion

Genomic studies on tadpole tail regression have revealed a number of modulated transcripts [5-9]. In comparison, our extensive proteomic analyses showed relatively few changes within the first 48 h of T_3 _treatment. This provides support for the hypothesis that changes in the transcriptome are the major target for the establishment of the tail regression program. However, this does not imply that the few changes in the (phospho)proteome during this time are not important, as these changes may support the transcriptome transition. For example, the tyrosine kinase inhibitor, genistein, prevents T_3_-dependent tail regression likely through inhibition of protein kinase C activity and TRα phosphorylation [16].

Our proteomic analyses may be limited in their sensitivity to reveal phosphorylation of low-abundance signaling phosphoproteins since no enrichment was performed. Nevertheless our data suggest that RLK I could be posttranslationally modified due to a T_3 _signal most likely through the early action of caspases thereby indicating an early apoptotic event. Our findings with GILT maturation, Ig heavy chains and cathepsin D increases show that the involvement of the immune system in the regressing tadpole tail may be greater than what has been generally considered. Furthermore, a large proportion of the transcripts identified by genomic studies encode transcription factors [5-9] whose protein products are most likely below the detection limits of our 2D PAGE and iTRAQ analyses. An improved fractionation strategy for the iTRAQ analysis may reveal these.

The present study provides important insights into the molecular mechanism of T_3_-dependent tadpole tail regression during postembryonic development which includes novel components at the proteome level.

## Methods

### Experimental animals

The care and treatment of animals used in this study were in accordance with the guidelines of the Animal Care Committee, University of Victoria, under the Canadian Council of Animal Care.

*Rana catesbeiana *tadpoles were locally caught (Victoria, BC) or purchased (Ward's Natural Science Ltd., St, Catharines, ON). Animals were housed in the University of Victoria aquatics facility and maintained in 360 L all-glass flow-through aquaria containing recirculated water at 15°C with exposure to natural daylight. Tadpoles were fed daily with spirulina (Aquatic ELO-Systems, Inc., FL). *R. catesbeiana *tadpoles, TK stage VI-VIII [31], were exposed to 10 nM T_3 _(80 μl of 10^-3 ^M T_3 _in 2.5 mM NaOH) (Sigma-Aldrich, St. Louis MO USA) or vehicle control (80 μl 2.5 mM NaOH) in the rearing water (8 L per 6 tadpoles) for 24 or 48 h. Tadpoles were acclimatized for 24 h prior to exposures to 24°C, and were not fed during this period or during exposures. For the 2D gel analysis, each treatment or control group consisted of 18 pooled tadpoles. The 48 hour 2D analyses consisted of three, completely independent, control and T_3 _treatment groups, while the 24 hour analyses were from a single control and T_3 _treatment group. The 24 hour analyses were only done to further characterize absence or presence of proteins observed to be altered at the 48 hour time point. For the iTRAQ analysis, two independent T_3_-treatment groups and two independent control groups consisting of six pooled tadpoles per group were used. Tadpoles were euthanized in 0.1% tricaine methanesulfonate (Syndel Laboratories, Vancouver, BC, Canada) in 25 mM sodium bicarbonate, dorsal and ventral tail fins were removed on ice, and proteins extracted as described below. For QPCR analysis, premetamorphic tadpoles (stage VI-VIII) were exposed to 100 nM T_3 _or vehicle control in the rearing water for 24, 48 and 72 h and euthanized as above. Tadpoles at the indicated stages were also collected to determine gene expression in tail tissue during natural metamorphosis.

### Subcellular fractionation

Two separate procedures were used for the isolation of nuclei and for the cytosolic-mitochondrial-microsomal fractions. All extraction procedures were performed on ice or at 4°C. Tail fin tissue was rinsed with extraction buffer lacking detergents or inhibitors (see below for composition). Three ml of buffer were used per gram of tissue, which was homogenized in a teflon-glass Dounce homogenizer (Wheaton, USA). For the nuclear isolation tissue was homogenized in 20 mM N-[2-Hydroxyethyl]piperazine-N'-[2-ethanosulfonic acid] (HEPES) pH 7.5, 0.25 M sucrose (ACP Chemicals Inc., Montreal Canada), 25 mM KCl, 5 mM MgCl_2 _(both from BDH chemicals, Toronto Canada), 0.5% Triton X-100, 1mM dithiothreitol (DTT), 2 mM vanadyl ribonucleoside complexes (VRCs), 100 μM phenylmethylsulfonyl fluoride (PMSF), 4 μg/ml aprotinin, 1 μg/ml leupeptin, 2 μg/ml antipain, 300 μg/ml benzamidine, 10 mM β-glycerophosphate, 0.2 mM Na_3_VO_4_, and 1 mM NaF (all remaining from Sigma-Aldrich).

Homogenization efficiency and integrity of nuclei was checked with microscopy and 4'-6-Diamidino-2-phenylindole (DAPI) staining for nuclei. The homogenate was incubated on ice with shaking for 30 min then passed through 100 μm nylon mesh cell-strainer. The homogenate was centrifuged at 1000 × g for 10 min. The nuclei were resuspended in the above extraction buffer (3 ml per g of initial tissue), layered onto a 0.88 M sucrose cushion, which contained everything the extraction buffer did except for Triton X-100 and VRCs, and centrifuged at 2000 × g for 30 min. The resulting nuclear pellet was washed in extraction buffer, without Triton X-100 and RVCs, and centrifuged at 2000 × g for 10 min. The nuclear pellet was stored at -70°C.

To obtain the cytosolic, mitochondrial and microsomal fractions, tail fin tissue was homogenized in 20 mM HEPES pH 7.5, 0.25 M sucrose, 5 mM Ethylenedinitrilo-tetraacetic acid (EDTA) (EM Science, Gibbstown NJ USA), 1mM DTT, 100 μM PMSF, 4 μg/ml aprotinin, 1 μg/ml leupeptin, 2 μg/ml antipain, 300 μg/ml benzamidine, 10 mM β-glycerophosphate, 0.2 mM Na_3_VO_4_, 1 mM NaF. The homogenate was centrifuged at 800 × g for 10 min. The supernatant was then centrifuged at 12,200 × g for 15 min. The pellet was washed twice in extraction buffer and centrifuged at 12,200 × g for 15 min. This mitochondrial pellet was stored at -70°C. The supernatant was centrifuged at 100,000 × g for 1 hour to obtain the microsomal pellet and cytosolic fractions which were then stored at -70°C.

### Anion-exchange HPLC fractionation of cytosolic fraction

The cytosolic fraction was first desalted using size-exclusion chromatography with Sephadex G-25-M beads (Sigma-Aldrich). Sample was kept at 4–8°C throughout the procedure. Six ml of sample was loaded onto a 27 ml column with 60 mM ammonium bicarbonate (EM Science) as buffer. The eluted protein fractions in the first 7.5 ml were kept, frozen at -70°C, and lyophilized for 48 h. The sample was reconstituted in 40 mM ammonium bicarbonate to a concentration of 10 mg/ml. Fractionation of the desalted cytosolic sample was performed using a Waters HPLC setup with a Waters 600S controller, 626 pump and 486 tunable absorbance detector (Waters, Milford MA USA). The column used consisted of 2 ml Accell Plus QMA 300Å anion-exchange media packed into a AP Minicolumn assembly (5 × 100 mm) (Waters). Five mg of total cytosolic sample was loaded onto the column in 40 mM ammonium bicarbonate (pH 8.3), the unbound proteins were captured as the 40 mM fraction. The remaining sample was fractionated using a step-gradient of 190, 260, 340 mM and 1 M ammonium bicarbonate. The flow rate was set at 1 ml/minute and each step was 13 min. The detector was set at 280 nm. The fractions were collected on ice, frozen at -70°C, and lyophilized for 48 h.

### 2D polyacrylamide gel electrophoresis

Proteins from the nuclear pellet were initially extracted in an isoelectric focusing (IEF) buffer lacking ampholytes consisting of 9.5 M urea (SigmaUltra), 4% 3-[(3-cholamidopropyl)dimethylammonio]-1-propanesulfonate (CHAPS), 1% DTT, 40 mM tris(hydroxymethyl)aminomethane (Tris-base) pH 8.5, 5 mM EDTA, 100 μM PMSF, 4 μg/ml aprotinin, 1 μg/ml leupeptin, 2 μg/ml antipain, 300 μg/ml benzamidine, 10 mM β-glycerophosphate, 0.2 mM Na_3_VO_4_, 1 mM NaF (all from Sigma-Aldrich) for 15 min at room temperature. DNA was then precipitated by adding 2% ampholytes (Pharmalyte 3–10 and 5–8 in a 2/3 and 1/3 ratio, respectively; Amersham Pharmacia Biotech AB, Uppsala Sweden), incubating for 15 min at room temperature, and pelleting the DNA at 12,000 × g for 10 min. Ampholytes were then replaced in the supernatent to 2% as indicated above. The mitochondrial and microsomal pellets and the lyophilized HPLC cytosolic fractions were all dissolved in the IEF buffer with the inclusion of 2% ampholytes as described above. Protein concentrations were assayed using the Bio-Rad Protein Assay (Hercules, CA USA) with protein standards dissolved in the IEF buffer. The amount of pellet to IEF buffer used yielded a protein extract with a protein concentration of 2–6 mg/ml.

Three hundred μg of protein for each fraction (except for the 40 mM cytosolic fraction where 100 μg of protein was used) were aliquoted, made up to 100 μl with IEF buffer, solubilized for seven hours at room temperature, centrifuged at 12,000 × g for 10 min, and loaded onto IEF tube gels used for the first dimension. The IEF tube gels (2.5 mm × 13 cm) consisted of 9.5 M urea, 3.897% acrylamide and 0.104% piperazine diacrylamide (wt/vol) (Bio-Rad), 2% CHAPS, 3.875% Pharmalyte 3–10 ampholytes, 2.375% Pharmalyte 5–8 ampholytes, and 0.05% ammonium persulphate (APS) (ACP Chemicals Inc.) and 0.07% *N,N,N*',*N*'-tetramethylethylenediamine (TEMED; Sigma-Aldrich) for polymerization.

The IEF gels were prefocused and run as described in [6] with the following modifications. Samples were electrophoresed for 16 h at 450 V, then hyperfocused at 800 V for 2 h (8800 Vh total). The IEF gels were then equilibrated using the same equilibration buffer as in [6] except the buffer contained 65 mM DTT for 15 min, and then for 15 min in 5 ml of IEF-equilibration buffer with 136 mM iodoacetamide (Sigma-Aldrich).

The IEF gels were then transferred onto the second dimension SDS-polyacrylamide gels (15 cm × 14 cm × 1.5 mm) comprised of an 11% separating gel (37.5:1 acrylamide to bis-acrylamide (Bio-Rad), 375 mM Tris-base pH 8.8, 0.1% SDS, 0.05% APS, 0.05% TEMED) and a 4% stacking gel (125 mM Tris-base pH 6.8, 0.1% SDS, 0.05% APS, 0.1% TEMED). The IEF gels were overlayed with 0.5% agarose (EM Science) in 125 mM Tris-HCl pH 6.8 with 0.05% bromophenol blue/2% SDS. Precision Plus Dual Color (Bio-Rad) molecular weight markers were used. The gels were electrophoresed at 30 mA per gel at constant current in SDS running buffer (25 mM Tris-base, 192 mM glycine (EM Science), 0.1% SDS) until the bromophenol blue ran out of the bottom of the gel,.

The 2D gels were then stained with a fluorescent phosphoprotein stain, ProQ Diamond, according to the manufacturer's instructions (Invitrogen-Molecular Probes, Burlington, Ontario, Canada). Fluorescence was detected with UV light using a ChemiImager 4000 imaging system (Alpha Innotech Corporation, San Leandro CA USA). The gels were then stained for total protein by washing in 50% ethanol (EM Science) and 3% phosphoric acid with shaking overnight, rinsing three times for 30 min in water, equilibrating in 16% ammonium sulfate (EM Science), 25% methanol (EM Science), 5% phosphoric acid for 1 hour, and subsequently staining by adding Coomassie brilliant blue G250 (Sigma-Aldrich) to 0.01% and shaking for 4 days. Images were obtained by scanning the gels with a Li-Cor Odyssey scanner (Li-Cor, Lincoln NE USA) at 700 nm. Protein spot density was analyzed using Li-Cor Odyssey Ver. 2.0.5 software (Li-Cor). Gel images were analyzed using Adobe PhotoShop Ver. 5.0 (Adobe Systems Inc., San Jose CA USA).

### Mass spectrometry analysis

Protein spots of interest were excised from the 2D gels and the proteins within were reduced, alkylated, and digested with trypsin according to an in-gel digestion protocol [100] with a few modifications. The gel pieces were destained in 50% methanol/5% acetic acid (ACP Chemicals Inc.), dehydrated with acetonitrile (EM Science), dried, reduced with 50 mM DTT in 100 mM ammonium bicarbonate at 56°C for 30 min, alkylated with 100 mM iodoacetamide in 100 mM ammonium bicarbonate at 45°C for 30 min, dehydrated with acetonitrile, hydrated with 100 mM ammonium bicarbonate, dehydrated with acetonitrile, dried, and digested with 20 ng/μl of sequencing grade modified trypsin (Promega Corp., Madison WI USA) in 50 mM ammonium bicarbonate at 37°C overnight. The resulting peptides were extracted out of the gel pieces by successive incubation in 50 mM ammonium bicarbonate for 1 hour at 37°C and twice in 0.1% formic acid (ACP Chemicals Inc.) for 30 min at 37°C. The peptides were desalted using ZipTip pipette tips containing C18 reversed-phase media (Millipore Corp., Bedford, MA USA) by washing with 0.1% formic acid and eluting with 75% acetonitrile/0.1% formic acid. Peptides were either analyzed by MALDI-TOF MS as follows or by ESI-QqTOF or MALDI-TOF-TOF MS analysis as described in the iTRAQ section. Peptide samples were applied to the target plate with an equal volume of matrix solution (1% α-cyano-4-hydroxycynnamic acid (Sigma-Aldrich) in 50% acetonitrile/0.3% formic acid) and allowed to dry. Adrenocorticotropic hormone fragment 1–17 (FW 2093.4), bradykinin fragment 2–9 (FW 904.0) and angiotensin 1 (FW 1296.5) (Sigma-Aldrich) were used as external calibrants.

Spectra were obtained using a Voyager-DE STR Biospectrometry Workstation MALDI-TOF mass spectrometer (PE Applied Biosystems, Foster City, CA USA) operating in positive reflector mode with delayed extraction. Data were manipulated using the Voyager Version 5.1 software with Data Explorer (PE Applied Biosystems). The sample spectra were further internally calibrated using autolytic trypsin peptide peaks. The masses of the tryptic peptides were used to search a local protein database using MASCOT Peptide Mass Fingerprint software (Matrix Science Inc., Boston MA USA) using the following settings: all entries, 1 missed cleavage, cysteines modified by carbamidomethylation, oxidized methionines, deamidation of asparagine and glutamine, peptide mass tolerance of ± 50 ppm. If the tryptic peptides were analyzed by ESI-QqTOF generating MS/MS spectra, manual *de novo *sequencing was performed viewing the spectra on AnalystQS Ver. 1.1 software (Applied Biosystems, Foster City, CA USA) or automatically using PEAKS Studio Ver.3.0 software (Bioinformatics Solutions Inc., Waterloo ON Canada) as explained in the data analyses section for iTRAQ. The group of peptide spectra that were obtained from the RLK I spot were analyzed using SPIDER software [101].

### Immunoblotting

Protein samples from the total extract and subcellular fractions were analyzed for lamin B1+B2 and for cytochrome c. Protein samples of the 48 hour control and treatment microsomal fraction were analyzed for cytokeratin in duplicate. Thirty μg of protein per sample was boiled for 3 min in 25 μl of 60 mM Tris-base pH 6.8, 2% SDS, 10% glycerol, 100 mM DTT, 0.03% bromophenol blue and loaded onto a 12% SDS-PAGE (see '2D gel electrophoresis' above for gel and buffer compositions). Proteins were then electrophoretically transferred onto a nitrocellulose membrane (Bio-Rad). Membranes were blocked with 5% nonfat milk in 140 mM NaCl/20 mM Tris-base pH 7.6 (TBS), for 1 hour, then probed with a pan-cytokeratin rabbit polyclonal IgG antibody (H-240; Santa Cruz Biotechnology Inc., Santa Cruz CA USA) diluted at 1/500 or an anti-lamin B1+B2 mouse monoclonal IgG1 antibody (ab4825; Cedarlane, Hornby ON Canada) diluted at 1/50 or anti-cytochrome c rabbit polyclonal antibody (H-104; Santa Cruz Biotechnology Inc.) diluted at 1/200 in 5% nonfat milk/TBS and 0.1% Tween (TBST) with gentle agitation for 1 hour at room temperature. The blots were washed with TBST for 1 hour. The membranes were then incubated with secondary antibody, IRDye-800 conjugated anti-rabbit IgG or IRDye-800 conjugated anti-mouse IgG (Rockland Inc., Gilbertsville PA USA) diluted at 1/2000 in 5% nonfat milk/TBST for 1 hour at room temperature. The blots were washed with TBST for 1 hour. The prepared blots were scanned by a Li-Cor Odyssey scanner at 800 nm.

### Isolation of RNA, generation of cDNA, degenerate-primer PCR and 5'-/3'-RACE

RNA was isolated and cDNA generated from premetamorphic tadpole tail fin tissue as described in Veldhoen et al [9]. The resulting cDNA was used for PCR using degenerate primers: forward: 5'-GAA/G GCA/C GCC AAT/C ACT/C GAA/G CT-3' and reverse: 5'-AC C/TTG T/AAG C/TTC CTC T/CTC G/ATG-3' (32 fold degeneracy each) with an initial 94°C for 10 min then 40 cycles at 94°C for 30 sec, 52°C for 30 sec, and 72°C for 30 sec, followed by 10 min at 72°C. This generated a single product at 380 bp. The PCR product was cloned using the TOPO TA Cloning Kit System with pCR2.1 TOPO vector and TOP10 cells (Invitrogen) as described previously [102]. The insert was then sequenced in both directions using the TOPO kit M13 forward and reverse primers at the University of Victoria sequencing facility. Based on this sequence two gene specific primers (GSP) were designed: GSP1 for 5'-RACE: 5'-TGTCTCCACCGCTAACACCAACGCCAAC-3' and GSP2 for 3'-RACE: 5'-GTTGGCGTTGGTGTTAGCGGTGGAGACA-3'. RACE-ready cDNA libraries were made from total RNA using the Smart RACE cDNA kit (BD Biosciences, Burlington ON Canada). The RACE reactions used the universal primers supplied with the kit paired with the GSP1 and GSP2. Touchdown PCR was performed on the RACE-ready libraries: 94°C for 2 min; five cycles at 94°C for 30 sec, and 72°C for 3 min; five cycles at 94°C for 30 sec, 70°C for 30 sec, and 72°C for 3 min; and 25 cycles at 94°C for 30 sec, 68°C for 30 sec, and 72°C for 3 min. The 5'-RACE produced a single product of ~600 bp and the 3' RACE produced three products between ~1400 and ~1500 bp. The PCR products were cloned as above and the longest clones from each RACE reaction were sequenced as above, giving two overlapping sequences that encoded the complete open reading frame. The sequence reported in this manuscript is accessible in GenBank [GenBank: EF156435].

### Quantitation of gene expression

The expression of RLK I gene transcript was quantified in tails of individual animals (n = 4 for all treatments and/or time points). Tail tissue treatment, RNA isolation and cDNA generation was performed as indicated above and expression data were collected using a real-time quantitative PCR assay on a MX4000 system (Stratagene, La Jolla, CA, USA) as described previously [103] using the following primers for RLK I: 5'-GTTGGCGTTGGTGTTAGCGG-3' and 5'-GGCACTGCTTCTTGCAACTTG-3'. The QPCR product from the keratin transcript was analyzed on a gel and sequenced ensuring that only this specific keratin transcript was being analyzed. The invariant L8 ribosomal protein transcript was used as a normalizer [103].

### Differential expression analysis using iTRAQ

#### Peptide preparation, iTRAQ labeling, two-dimensional liquid chromatography separation and MS analysis

Proteins from tadpole tail fin tissue were extracted by homogenization in 6 M urea, 0.2% SDS, 20 mM HEPES pH 7.5 (3 ml/g tissue). The homogenate was centrifuged at 12,000 × g for 15 min at 4°C. The supernatant was adjusted to 4 M urea, 0.05% SDS, 2 mM MgCl_2 _and 20 mM HEPES pH 8.0, and DNA was digested with 10 U/ml of benzonase for 30 min at 8°C. The iTRAQ reagents were used according to the manufacturer's protocol with minor modifications as follows (Applied Biosystems).

Two independent control and two independent treatment protein samples were quantified using the bicinchoninic acid assay (BCA) (Sigma-Aldrich, St Louis, MO USA). One hundred μg of each sample (~55 μl) was precipitated in 1 ml cold acetone, overnight at -20°C. Proteins were then reduced in 3.3 mM tris-(2-carboxyethyl) phosphine, alkylated with 6.7 mM methyl methane thiosulfonate (MMTS) and digested overnight with 10 μg of sequencing grade modified trypsin (Promega, Madison, WI USA) at 37°C. The resulting peptides from the two control samples were labeled with iTRAQ reagents 114 and 116, while peptides from the two T_3 _treatment samples were labeled with iTRAQ reagents 115 and 117.

The labeled peptide samples were then pooled, adjusted to pH 2.5–3.0 with concentrated phosphoric acid (ACP Chemicals Inc., Montreal, QC Canada), and separated by strong cation-exchange chromatography (SCX) using a PolySULFOETHYL A SCX column (100 × 4.6 mm, 5μm, 300Å - PolyLC Inc., Columbia, MD USA) on the VISION Workstation (Applied Biosystems, Foster City, CA USA). Mobile phases used were: Buffer A: 10 mM monobasic potassium phosphate (Sigma-Aldrich, St Louis, MO, USA), 25% acetonitrile (ACN) (EMD Chemicals, Gibbstown, NJ, USA), pH 2.7 and Buffer B: same as A with the addition of 0.5 M potassium chloride (Sigma-Aldrich St Louis, MO, USA). Fractions of 500 μL were collected over an 80 minute gradient: 0–30 min, 5% to 35% Buffer B; 30–80 min, 35% to 100% Buffer B. Sixteen SCX fractions were reduced to 150 μL using a SpeedVac and subjected to reverse-phase chromatography using an integrated system consisting of a Famos autosampler, Switchos switching pump, UltiMate micro pump and a Probot microfraction collector (LC Packings, Amsterdam, Netherlands). The SCX fractions were first desalted on a C18 PepMap guard column (300 μm i.d. x 5 mm 5 μm, 100Å, LC Packings, Amsterdam) at 50 μl/minute flow rate for 15 min. Peptides were separated over a manually packed 75 μm × 15 cm C18 column (Magic C18Aq, 5μm, 100Å, Michrom Bioresources Inc., Auburn CA, USA) using a 85 min gradient of 5%-75% ACN in 0.1% formic acid flowing at 250 nL/min.

The eluant was either sprayed directly into an Applied Biosystems QSTAR Pulsar i (Applied Biosystems/MDS SCIEX Concord, ON Canada) (ESI-QqTOF) mass spectrometer or spotted onto a MALDI target plate by the Probot for LC-MALDI analysis. The QSTAR operating software Analyst QS v1.1 employed an information dependent acquisition (IDA) method for optimized MSMS spectra acquisition over a 6 sec cycle which was repeated over the duration of the gradient. The MS-TOF survey scan lasted for 1 sec over a range of 400–1200 m/z targeting precursor ions of charge state 2–5+ that exceeded a threshold of 20 counts. Former target ions within 100 ppm were excluded for the next 180 sec. The two most intense ions that met the IDA criteria were gated and fragmented with optimized collision energy. Each product ion scan lasted for 2.5 sec over a range of 100–1500 m/z. 'Enhance-all' was turned on for the product ion scans. LC-MALDI data was acquired on an Applied Biosystems 4800 MALDI TOF/TOF Analyzer (Applied Biosystems/MDS Sciex Foster City, CA USA). Sample spots were overlaid with 3 mg/mL α-cyano-4-hydroxycinnamic acid (CHCA) (Sigma-Aldrich, St Louis, MO USA) matrix in 50% ACN and 0.1% TFA. MS data was automatically acquired over a mass range of 800–4000 Da using fixed laser intensity for 500 shots with a uniformly random spot search pattern. In each spot, the 30 strongest peaks by MS (determined by cluster area) were selected for MS/MS using a job-wide interpretation method which excludes ions with a signal-to-noise of less than 60 and filters identical peaks detected in multiple spots. A 1 KV positive MS/MS operating mode was used with the relative precursor mass window set at 150 and collision induced dissociation turned on. MALDI-TOF-TOF data analysis was performed using GPS Explorer Ver. 3.0 (Applied Biosystems) software using the MASCOT (Matrix Science) search engine for protein database searching.

#### iTRAQ data analysis

All of the MS data in ProQUANT (Applied Biosystems) resulting from the ESI-QqTOF analysis was transferred to Excel (Microsoft Corporation, Redmond WA) where the data was filtered to reveal only those MS spectra where a change of 1.5 fold or higher was observed between the treatment and the control for both duplicates. An additional requirement was that the ratio of the iTRAQ tags between the two controls had to be between 0.67 and 1.5. The resulting MS spectra were then manually inspected in AnalystQS 1.1 (Applied Biosystems) software to ensure good quality of the iTRAQ tags and fragmentation information.

High quality spectra where then used for *de novo *peptide sequencing. Peptide sequence was obtained by either manually interpreting spectra and/or through the use of PEAKS Studio Ver.3.0 (Bioinformatics Solutions Inc.) software capable of auto *de novo *sequencing. The parameters were set as follows: parent mass error tolerance of 0.1 Da, fragment mass error tolerance of 0.1 Da, enzyme used was trypsin, fixed posttranslational modifications (PTMs) were MMTS on cysteines, iTRAQ tags on N-terminal and lysine, and variable PTMs were iTRAQ tag on tyrosine and oxidation of methionine histidine and tryptophan. All of the results were manually inspected to ensure correctness. The resulting peptide sequences were then queried against the Entrez Protein (NCBInr) database (Oct. 15, 2006) using BLASTp [21] to allow for short, nearly exact matches: no compositional adjustments, a low complexity filter, expect threshold of 20000, word size of 2, using matrix PAM 30, with gap costs of 9 for existence, and 1 for extension, searching all metazoan sequences.

### Statistical analyses

Statistical analyses were performed using SPSS Ver. 12.0. (Chicago IL USA). For the 48 hour 2D gel analysis data P-values were determined using a one-way ANOVA with a test for homogeneity of variance and a Shapiro-Wilk test of normality. For the QPCR analyses P-values were determined using the Mann-Whitney U non-parametric two-tailed test.

## Authors' contributions

DD participated in the study design and performed the experiments and analyses. CCH conceived of the study, and participated in its design and analyses. Both authors participated in the writing of the manuscript. All authors read and approved the final manuscript.

## Supplementary Material

Additional file 1 Additional changes identified in 2D analyses of the microsomal fraction. 2D gel regions of the microsomal fraction showing the increase of a protein spot at ~30 kDa and pI ~5 due to T_3 _treatment at 48 h. Relative molecular weights of protein standards are indicated in kDa. Spot density measurements (in arbitrary values) are graphed for the corresponding 2D gels on the left. The white bar represents the control while the gray bar represents the T_3 _treatment. Error bars represent the standard error of the mean from three independent controls and three independent T_3 _samples. Significance is indicated by an asterisk for p < 0.05 (ANOVA). Values adjacent to the gray bar represent the fold increase due to T_3_. Spot density measurements were normalized between the gels with the β-actin protein spot. ESI-QqTOF MS analysis of the protein spot is shown in Additional file 2. Click here for file

Additional file 2 MS analysis of protein spot changing in the microsomal fraction. Table Click here for file
