# Supplementary figures and images for: Analysis of the Rana catesbeiana tadpole tail fin proteome and phosphoproteome during T3-induced apoptosis: identification of a novel type I keratin
Source: BMC Dev Biol. 2007 Aug 6;7:94. doi: 10.1186/1471-213X-7-94 (PMC2025591; doi:10.1186/1471-213X-7-94)

## Slide 1
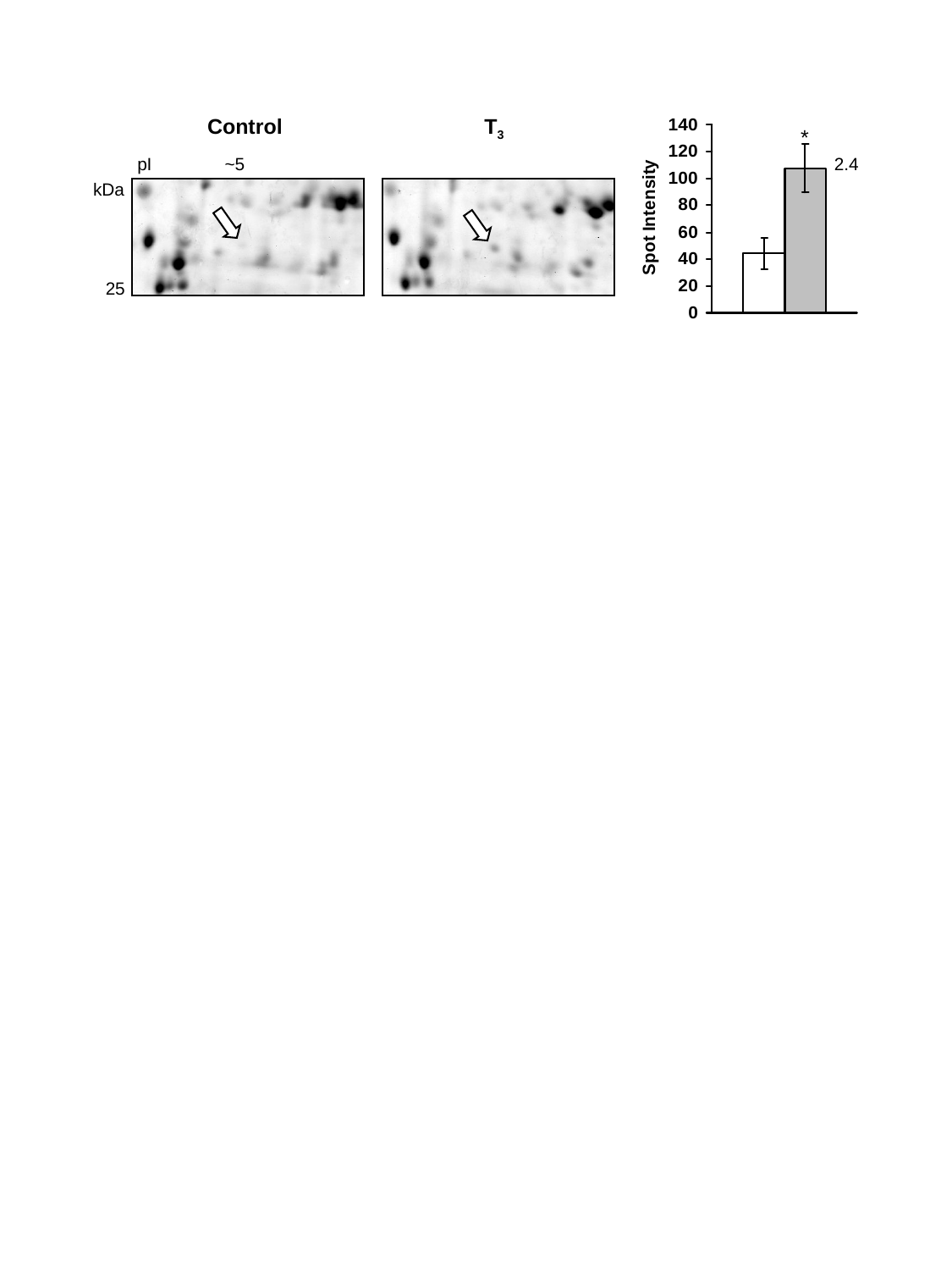

Control
T3
*
pI ~5
2.4
kDa
25

Supplement: Additional file 1 — Additional changes identified in 2D analyses of the microsomal fraction. 2D gel regions of the microsomal fraction showing the increase of a protein spot at ~30 kDa and pI ~5 due to T3 treatment at 48 h. Relative molecular weights of protein standards are indicated in kDa. Spot density measurements (in arbitrary values) are graphed for the corresponding 2D gels on the left. The white bar represents the control while the gray bar represents the T3 treatment. Error bars represent the standard error of the mean from three independent controls and three independent T3 samples. Significance is indicated by an asterisk for p < 0.05 (ANOVA). Values adjacent to the gray bar represent the fold increase due to T3. Spot density measurements were normalized between the gels with the β-actin protein spot. ESI-QqTOF MS analysis of the protein spot is shown in Additional file 2. [file 1471-213X-7-94-S1.ppt]
