# Supplementary material for: Analysis of the Rana catesbeiana tadpole tail fin proteome and phosphoproteome during T3-induced apoptosis: identification of a novel type I keratin
Source: BMC Dev Biol. 2007 Aug 6;7:94. doi: 10.1186/1471-213X-7-94 (PMC2025591; doi:10.1186/1471-213X-7-94)
Supplement: Additional file 2 — MS analysis of protein spot changing in the microsomal fraction. Table [file 1471-213X-7-94-S2.doc]

**Additional file 2. MS analysis of protein spot changing in the microsomal fraction**

^1^Observed peptide masses resulting from the tryptic digestion of the protein spot, reported as singly charged. ^2^Peptide sequence information deduced from MS/MS spectra of the corresponding peptides. ^3^Indicates that sequence could only be obtained for a fraction of the observed peptide. The masses of isoleucine are indistinguishable from leucine in MS and therefore L can be I and *vice versa*. ^4^Percent confidence for the peptide sequences as reported by PEAKS software.
